# Diet and Macronutrient Optimization in Wild Ursids: A Comparison of Grizzly Bears with Sympatric and Allopatric Black Bears

**DOI:** 10.1371/journal.pone.0153702

**Published:** 2016-05-18

**Authors:** Cecily M. Costello, Steven L. Cain, Shannon Pils, Leslie Frattaroli, Mark A. Haroldson, Frank T. van Manen

**Affiliations:** 1 College of Forestry and Conservation, University of Montana, Missoula, Montana, United States of America; 2 Grand Teton National Park, Moose, Wyoming, United States of America; 3 U. S. Geological Survey, Northern Rocky Mountain Science Center, Interagency Grizzly Bear Study Team, Bozeman, Montana, United States of America; Sonoma State University, UNITED STATES

## Abstract

When fed ad libitum, ursids can maximize mass gain by selecting mixed diets wherein protein provides 17 ± 4% of digestible energy, relative to carbohydrates or lipids. In the wild, this ability is likely constrained by seasonal food availability, limits of intake rate as body size increases, and competition. By visiting locations of 37 individuals during 274 bear-days, we documented foods consumed by grizzly (*Ursus arctos*) and black bears (*Ursus americanus*) in Grand Teton National Park during 2004–2006. Based on published nutritional data, we estimated foods and macronutrients as percentages of daily energy intake. Using principal components and cluster analyses, we identified 14 daily diet types. Only 4 diets, accounting for 21% of days, provided protein levels within the optimal range. Nine diets (75% of days) led to over-consumption of protein, and 1 diet (3% of days) led to under-consumption. Highest protein levels were associated with animal matter (i.e., insects, vertebrates), which accounted for 46–47% of daily energy for both species. As predicted: 1) daily diets dominated by high-energy vertebrates were positively associated with grizzly bears and mean percent protein intake was positively associated with body mass; 2) diets dominated by low-protein fruits were positively associated with smaller-bodied black bears; and 3) mean protein was highest during spring, when high-energy plant foods were scarce, however it was also higher than optimal during summer and fall. Contrary to our prediction: 4) allopatric black bears did not exhibit food selection for high-energy foods similar to grizzly bears. Although optimal gain of body mass was typically constrained, bears usually opted for the energetically superior trade-off of consuming high-energy, high-protein foods. Given protein digestion efficiency similar to obligate carnivores, this choice likely supported mass gain, consistent with studies showing monthly increases in percent body fat among bears in this region.

## Introduction

Laboratory studies (e.g., [1]) have demonstrated that many animals are capable of adjusting their selection of foods and the amounts eaten to regulate the intake of macronutrients (protein, carbohydrates, and lipids) and maximize dietary efficiency and growth. Only a few studies (e.g., [2]), largely involving primates, have directly measured macronutrient balance in the diets of wild animals, and to date, none have done so for wild vertebrate carnivores [3]. Unlike many of their strictly carnivorous relatives within the Carnivora, most bear species are opportunistic omnivores, often with a substantial proportion of plant foods in their diet. Bears are known to alter their diet in response to seasonal changes in food availability, often resulting from changes in plant phenology and prey vulnerability. Seasonal variation in food selection is probably most profound for bear species residing in northern regions that have evolved to hibernate in response to the lack of food availability during winter. During fall, these bears often devote nearly all their activity to finding and consuming high-quality foods, because they must accumulate adequate fat stores to sustain them for hibernating periods of up to 6 months [4]. Large body size also confers a reproductive advantage to both females [5] and males [6]. Thus, among carnivores, bears may be uniquely adapted to “over-eat” and to efficiently convert excess calories to lean body mass and body fat [7]. Recent studies on bears in captivity have demonstrated that bears are energy maximizers, consuming 8–9 times the digestible energy/day compared with the carnivore basal metabolic rate when offered food ad libitum [7, 8]. But, they have also shown that bears forego strict energy maximization to optimize their intake of macronutrients. When a variety of foods are available, bears do not feed exclusively on the most energy-rich foods, but consume mixtures of foods in which protein provides approximately 17 ± 4% of digestible energy (or 22 ± 6% of dry matter) relative to carbohydrates and/or lipids. This optimal protein level maximizes their mass gain per unit of energy intake [8]. Although bears prefer lipids over carbohydrates, both of these energy sources can be efficiently utilized to optimize the protein content of the diet.

In the wild, there are likely constraints on a bear’s ability to select foods necessary to maximize energy consumption and balance macronutrients. Foremost is seasonal variation in food availability. For example, with the exception of winter-killed ungulates, vertebrate prey, and over-wintered mast, most foods available to bears in the spring are plant foods of only low to moderate digestibility, providing limited opportunity for bears to maximize energy intake [9, 10]. Although black bears can gain weight on a spring diet dominated by graminoids and forbs [11], studies in captivity have shown that larger bears can be constrained from achieving mass gain when feeding exclusively on vegetation [12] or even fruit [13] due to the limitations of intake rate, bite size, and the physiological capacity of the gastrointestinal tract. For the larger-bodied grizzly bear, this constraint may limit body size and reproduction in regions where high-energy foods are absent, such as southeastern British Columbia [14]. Limits on the variety of foods available may also constrain bears from achieving the optimal protein balance. Using published data on bear foods, their seasonal availability, and their nutritional composition, Coogan et al. [15] quantified macronutrient content of potential diets of grizzly bears in Alberta. They estimated that bears were likely able to dilute protein intake to the optimal level by mixing carbohydrate-rich fruit with an otherwise high-protein diet during summer and fall, but that bears likely consumed surplus protein during the period prior to the onset of fruiting.

Competition from other bears is likely another constraint on food acquisition for smaller, subordinate individuals, or for females accompanied by young. Avoidance of large males by females and smaller males may reduce their ability to forage in areas of high food abundance, such as oak stands [16], and fish spawning streams [17]. Where two bear species coexist, interspecific competition also likely plays a role. Black bears often avoid the most productive salmon runs, where grizzly bears are dominant [18]. Larger adults are able to usurp and defend ungulate carcasses from smaller bears [19].

Although grizzly and black bears have similar dietary efficiencies [20] and feed on many of the same foods [9, 10], certain physical characteristics and behavioral adaptations give one species an advantage over the other in acquisition of particular foods. For example, with their short, curved foreclaws, black bears are capable of climbing trees to obtain fruits and nuts before they fall to the ground, whereas adult grizzly bears lack this climbing ability. Conversely, with their long foreclaws, grizzly bears are especially adapted for digging up tubers, corms, and subterranean rodents compared with black bears. Grizzly bears are also more adapted to carnivory, with larger body size, augmented shoulder musculature, and a more powerful bite [21].

We documented food selection by individual grizzly bears and black bears, monitored using global positioning system (GPS) technology, in Grand Teton National Park (GTNP) and vicinity during 2004–2006. Field investigations of GPS bear locations obtained during recent 24-hr periods afforded us the opportunity to quantify relative consumption of foods and macronutrients within daily diets based on feeding sign and scat analysis. At the time of this study, grizzly bears had recolonized only the northern half of GTNP [22], which provided us with a unique opportunity to examine differences in diets among grizzly bears, black bears that were sympatric with grizzly bears, and allopatric black bears. The delineation between sympatric and allopatric black bears was verifiable, because the temporal expansion of this grizzly bear population has been well documented [22, 23].

Our primary goals for this study were to compare seasonal food selection by GPS-collared grizzly and black bears and to examine whether bears were successful in optimizing macronutrient intake. We hypothesized that, on a daily basis, bears would select mixed diets designed to optimally balance macronutrients, but would be constrained by body size, season, and competition. We predicted that: 1) larger-bodied bears would include more high-energy foods (i.e., vertebrate prey [20]) in their daily diet than would smaller-bodied bears, thus proportion of protein in the daily diet would increase with body size, would be higher among grizzly bears than black bears, and would be higher among males than females; 2) smaller-bodied bears would be more likely to feed exclusively on vegetation or fruits than larger-bodied bears, thus frequency of daily diets with sub-optimal protein levels would decrease with body size, be higher among black bears than grizzly bears, and be higher among females than males; 3) bears would successfully balance macronutrients during summer and fall when carbohydrate- and lipid-rich foods were available, but would be constrained from doing so during spring due to the low availability of non-protein energy sources; and 4) due to the absence of interspecific competition and the presence of intraspecific competition, food selection by male and female allopatric black bears would be more similar to that of male and female grizzly bears, than that of male and female sympatric black bears, respectively.

## Materials and Methods

### Study area

**Fig 1:**
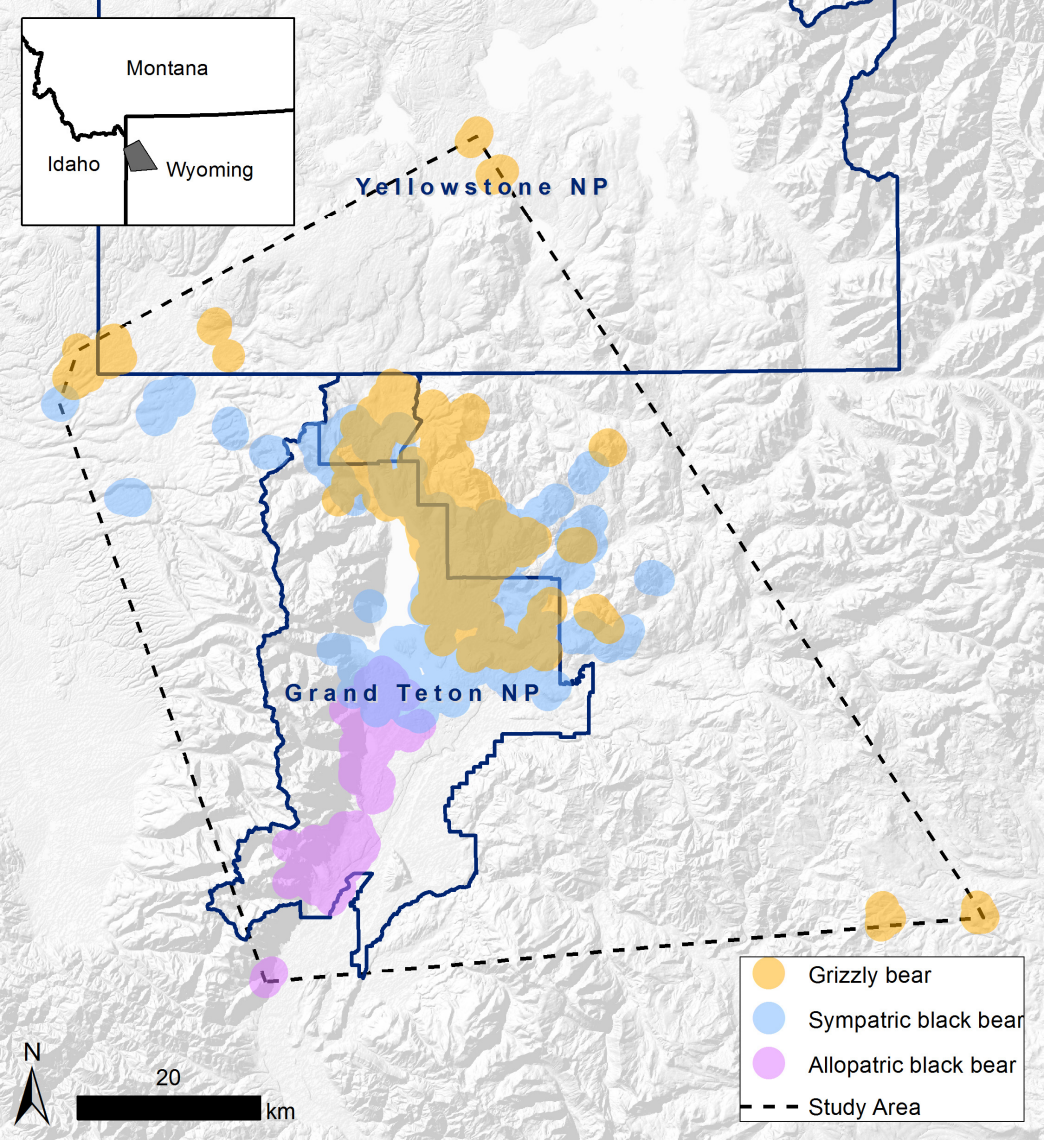
The study area centered on Grand Teton National Park, Wyoming, USA, as defined by the spatial extent of all site visits during 2004–2006. Colored areas depict locations of site visits by species: grizzly bear (yellow); sympatric black bear (blue); and allopatric black bear (pink).

Our study area, as defined by the spatial extent of bear location site visits, was approximately 5,500 km^2^ centered on GTNP (Fig 1), but included adjacent areas of the Bridger-Teton and Caribou-Targhee National Forests, Yellowstone National Park, and the John D. Rockefeller, Jr. Memorial Parkway. The area included several mountain ranges and riparian valleys of the upper Snake River drainage, with elevations ranging from 1,890 m to 4,197 m. Vegetation varied by elevation, with grasslands (*Poa*, *Festuca*, and *Calamagrostis* spp.), sagebrush (*Artemisia* spp.) shrublands, and riparian cottonwood (*Populus* spp.)-blue spruce (*Picea pungens*) forests occupying valley floors. Douglas-fir (*Pseudotsuga menziesii*), lodgepole pine (*Pinus contorta*), limber pine (*Pinus flexilis*), and quaking aspen (*Populus tremuloides*) forests dominated lower drier sites. Subalpine forests consisted of lodgepole pine, subalpine fir (*Abies lasiocarpa*), and Engelmann spruce (*P*. *engelmannii*). Whitebark pine (*P*. *albicaulis*) occurred at higher elevations to treeline. Alpine communities of sedge (*Carex* spp.) and forbs were interspersed among scree and talus slopes at the highest elevations. Vegetative communities and bear foods available were similar between the areas occupied by sympatric and allopatric bear populations. Climate was characterized by long, cold, snowy winters and short, cool summers. Mean 30-year (1981–2010) January and July temperatures were -10.2° and 15.7–16.6°C at Moran and Moose, respectively [24]. Mean annual precipitation was 554–626 mm, with the greatest seasonal precipitation occurring during winter as snow.

### Field methods

Our analysis draws on data obtained from two separate studies with similar methodologies. The study of sympatric black and grizzly bears was conducted June 2004–October 2006 and the study of allopatric black bears was conducted June 2005–September 2006. Bear capture and handling procedures used for this study were described in Schwartz et al. [25], and were approved by the Animal Care and Use Committee of the U.S. Geological Survey, Biological Resources Division, and conformed to the Animal Welfare Act and U.S. Government principles for the utilization and care of vertebrate animals used in testing, research, and training. All field work was conducted under permits issued by the U.S. Fish and Wildlife Service, the State of Wyoming, and the National Park Service.

Independent bears (≥3 years old) were fitted with Spread Spectrum GPS collars (Telonics, Inc., Mesa, Arizona) with an independent VHF transmitter, a CR-2a programmable breakaway collar release, a biodegradable canvas spacer; and a mortality sensor with a 4–5 hour delay. GPS transmitters were programmed to maximize the number of fixes over the duration of deployment for each collar depending on collar size: fix intervals varied from 35 to 190 minutes with shorter intervals associated with larger grizzly bears. Units were programmed to turn off during the denning season (31 Oct or 15 Nov to 14 Apr). Transmitters were programmed for time-specific data uploads, enabling us to retrieve GPS locations weekly by aircraft and conduct site visits while signs of bear foraging were still fresh. Each week during May to October, we randomly assigned a number to each bear, and a number to each day of the week for each bear. Sampled individuals started with the lowest-ranked bear on its lowest-ranked day of week, and continued through the list of ranked bears in an effort to sample as many bear-days as possible. For each bear-day, we visited all successful GPS locations collected for a 24-hour period, except for those bears with fix intervals of <1 hour, for which we visited at least one GPS fix each hour. Within 20 m of each location, we searched for scats and evidence of feeding activity (e.g., grazing, excavations, carcass remains). Observations of feeding sign were classified according to 3 levels of intensity: 1 (light), 2 (moderate), and 3 (heavy; S1 Appendix). Scats found at each site were collected and frozen. For analysis, we rinsed scats through coarse (3.36 mm) and fine (0.841 mm) sieves, identified food items to the finest possible taxonomic level, and estimated percent composition of each item [26]. When possible, mammal hair was identified to species using Moore et al. [27].

### Data analyses

We combined information from scats and feeding sign to quantify the relative contribution of different food types and macronutrients to a “daily” diet, recognizing that the temporal offset between items identified through scats versus feeding sign might sometimes result in a sample of foods consumed over a period exceeding 1 day by a few hours due to gut retention time. We considered each scat a unique sample unit. Due to differential digestion of various foods, we applied correction factors [28, 29] to estimate percent dietary volume from percent fecal volume within each scat (Table 1). For each food identified from feeding sign, we assigned a “scat equivalent” value based on the intensity of use assigned to that food during the site visit: light = 5%, moderate = 30%, and heavy = 75%. In other words, if an item was heavily consumed, we assumed it would be the equivalent of 75% of a scat in dietary (i.e., corrected) volume. These values were based on observed percent dietary volumes within scats, analyzed by each food type, and then summarized across food types (S1 Appendix). Summarizing the corrected scat volumes (from collected scats) and scat equivalents (from foods identified from feeding sign), we were able to estimate the percent contribution of each food item to the sampled dietary intake for each bear-day.

**Table 1:** Scat correction factors and food-specific energy values applied to food items identified in scats or from observation of feeding sign at site visits of grizzly and black bear locations, Grand Teton National Park and vicinity, Wyoming, USA, 2004–2006. Total energy was calculated as the mean of individual foods identified to genus or higher taxonomic group (e.g., Formicidae [S1 Table]).

	Scat correction factor	Energy values (kcal/g)^f^			Mean total energy (kcal/g)	
Food type		Protein	Carbohydrate	Lipid	Dry basis	Fresh basis
AGVEG^a^	0.3^c^	3.47	4.07	8.37	2.43	0.98
BGVEG^b^	0.8^c^	2.78	4.03	8.37	2.49	1.29
Cambium	0.4^e^	3.47	4.07	8.37	2.46	0.24
Fruit	1.0^c^	3.36	3.60	8.37	2.96	1.43
Nut	1.5^c^	3.47	4.07	8.37	3.35	3.15
Fungi	1.0^e^	2.62	4.07	8.37	2.96	0.31
Insect	1.1^c^	4.27	3.82	9.03	4.74	2.77
Vertebrate (small)	1.5^d^	4.27	3.82	9.03	4.61	1.27
Vertebrate (large)	2.0^d^	4.27	3.82	9.03	5.25	1.52

^a^ Above-ground vegetation

^b^ Below-ground vegetation

^c^ Hewitt and Robbins 1996

^d^ Persson et al. 2000

^e^ Extrapolated from above based on non-digestible content

^f^ Merrill and Watt 1973

We found 50 publications with data on the nutritional composition of foods identical or similar (i.e., within the same genera or other taxonomic group) to those consumed by bears during the study (S1 Table). We used these data to estimate the mean percentage of digestible protein, carbohydrate, and lipid, as well as the mean percentage of non-digestible content (e.g., ash, fiber) within each food identified, by genera or other taxonomic group (e.g., Formicidae [ants]). Studies differed in reporting, most notably in that some reported content of fresh foods (including moisture content), whereas others reported content on a dry matter basis. To facilitate direct comparison among food items and account for water content consumed, we converted dry matter contents to approximate fresh food content by utilizing either published values for moisture content or mean values for similar food types. In addition, many studies provided only percentage of protein or some other incomplete combination of constituents. For missing constituents, we extrapolated values based on mean relative values for similar foods. Finally, we assigned mean values for all constituents, by food type, when we could not find specific information about its genera or taxa.

We applied food-specific energy values for each of the macronutrients using Atwater specific factors [30] (Table 1). For each food item, we multiplied total units consumed/day (sum of scat and scat-equivalent percentages) × the percentage of each macronutrient within that food (fresh content) × the food-specific energy value associated with each macronutrient (kcal/g). Total energy was the sum of all of these values. Foods were then divided into 8 types: 1) above-ground vegetation ([AGVEG]; i.e., foliage, stems, flowers), 2) below-ground vegetation ([BGVEG]; i.e., roots, tubers, corms), 3) cambium, 4) fruit, 5) nut, 6) insect, 7) vertebrate, and 8) fungi. By summarizing for each of the 8 food types and each macronutrient, and dividing by total energy, we estimated the relative percentage of the daily digestible energy provided by each.

Pooling all bear-days, we performed principal components analysis (PCA) and hierarchical clustering on principal components (HCPC) using the FactoMineR package [31] for R (https://www.r-project.org) to cluster daily diets with similar food composition and assess associations of these daily diets with supplementary variables. Active variables used to construct the principal components were the relative daily percentages of total energy obtained from 7 food types (fungi were omitted due to extremely low values). Supplementary variables (i.e., those not used in the construction of the principal components, but useful to enrich interpretation) were: species (grizzly, sympatric black, and allopatric black); sex; season (spring [1 May–30 Jun], summer [1 Jul–20 Aug], and fall [21 Aug–31 Oct]), bear body mass at capture (Table 2 [kg]), and GPS fix interval. In HCPC, the selection of the number of clusters is subjective and Fransen et al. [32] recommended that the rationale for the decision be reported. We selected the minimum number of clusters that included homogenous diets for all food types that comprised ≥95% of energy for ≥1 day (i.e., diets dominated by one food type). A diet was considered homogenous when the food type accounted for at least 50% of daily energy during all bear-days included in the cluster and averaged ≥75% for the cluster.

**Table 2:** Body mass range (kg) and sample size (bear-days) for sex and species categories of bears monitored to estimate daily diets, Grand Teton National Park, Wyoming, USA, 2004–2006.

		*n* (individuals)	Body mass at capture (kg)	*n* (bear-days)			
Population	Sex			Spring	Summer	Fall	Total
Grizzly bear	F	4	104–147	9	10	21	40
	M	6	102–215	10	7	13	30
		10		19	17	34	70
Black bear	F	8	44–97	24	43	25	92
(sympatric)	M	9	80–149	18	21	13	52
		17		42	64	38	144
Black bear	F	5	55–77	10	17	16	43
(allopatric)	M	5	64–116	7	7	3	17
		10		17	24	19	60
Total		37		78	105	91	274

Using mixed-effects linear regression models with interactions (“lme” function in “nlme” package for R [33]), we tested for differences in percentage of protein in daily digestible energy as a function of season and species-sex categories, and as a function of season and bear body mass irrespective of species.

## Results

We obtained foraging data for 37 individuals during 274 bear-days (Table 2). Per bear-day, mean number of scats collected was 2.6 (range 0–13), mean number of observations of feeding sign was 8.2 (range 0–47), and mean number of samples (scats + scat equivalents) was 4.55 (range 0.05–17.00). We identified 134 different foods, with 115 foods identified to genus or species and 19 foods identified to a higher taxonomic group. Bears consumed AGVEG of 3 fern allies, 54 forbs, 16 graminoids, 3 shrubs, and 1 tree. The most frequently consumed foods in this category included licorice-root (*Ligusticum* spp.), sticky geranium (*Geranium viscocissum*), cow-parsnip (*Heracleum maximum*), dandelion (*Taraxacum* spp.), brome grass (*Bromus* spp.), sedge (*Carex* spp.), lousewort (*Pedicularis* spp.), bluegrass (*Poa* spp.), fireweed (*Epilobium* spp.), horsetails (*Equisetum* spp.), clover (*Trifolium* spp.), and reed grass (*Calamagrostis* spp.). Bears ate BGVEG of 1 aquatic, 5 forbs, and 1 graminoid, including yampa (*Perideridia* spp.), onion grass (*Melica* spp.), biscuitroot (*Lomatium* spp.), spring beauty (*Claytonia* spp.), bistort (*Polygonum* spp.), and pondweed (*Potamogeton* spp.). Five tree species provided cambium, but lodgepole pine was most frequently consumed. Bears consumed fruit of 21 shrubs, most frequently huckleberries (*Vaccinium* spp.), buffaloberries (*Shepherdia canadensis*), serviceberries (*Amelanchier alnifolia*), gooseberries (*Ribes* spp.), mountain-ash berries (*Sorbus scopulina*), hawthorn berries (*Crataegus* spp.), chokecherries (*Prunus virginiana*), and rose-hips (*Rosa* spp.). The nut category was represented by a single tree, whitebark pine (*Pinus albicaulis*). Insect foods included ants, wasps (Vespidae), unidentified insects, and unidentified larva. Eighteen vertebrate foods were observed, including large mammals, small mammals, birds, and bird eggs. Elk (*Cervus elaphus*) were, by far, the most frequently consumed vertebrate, followed by deer (*Odocoileus* spp.), domestic cattle (*Bos taurus*), and moose (*Alces alces*). The fungi category was represented by unidentified mushrooms. Bears consumed 1–20 different foods/day with an average of 7.3.

**Fig 2:**
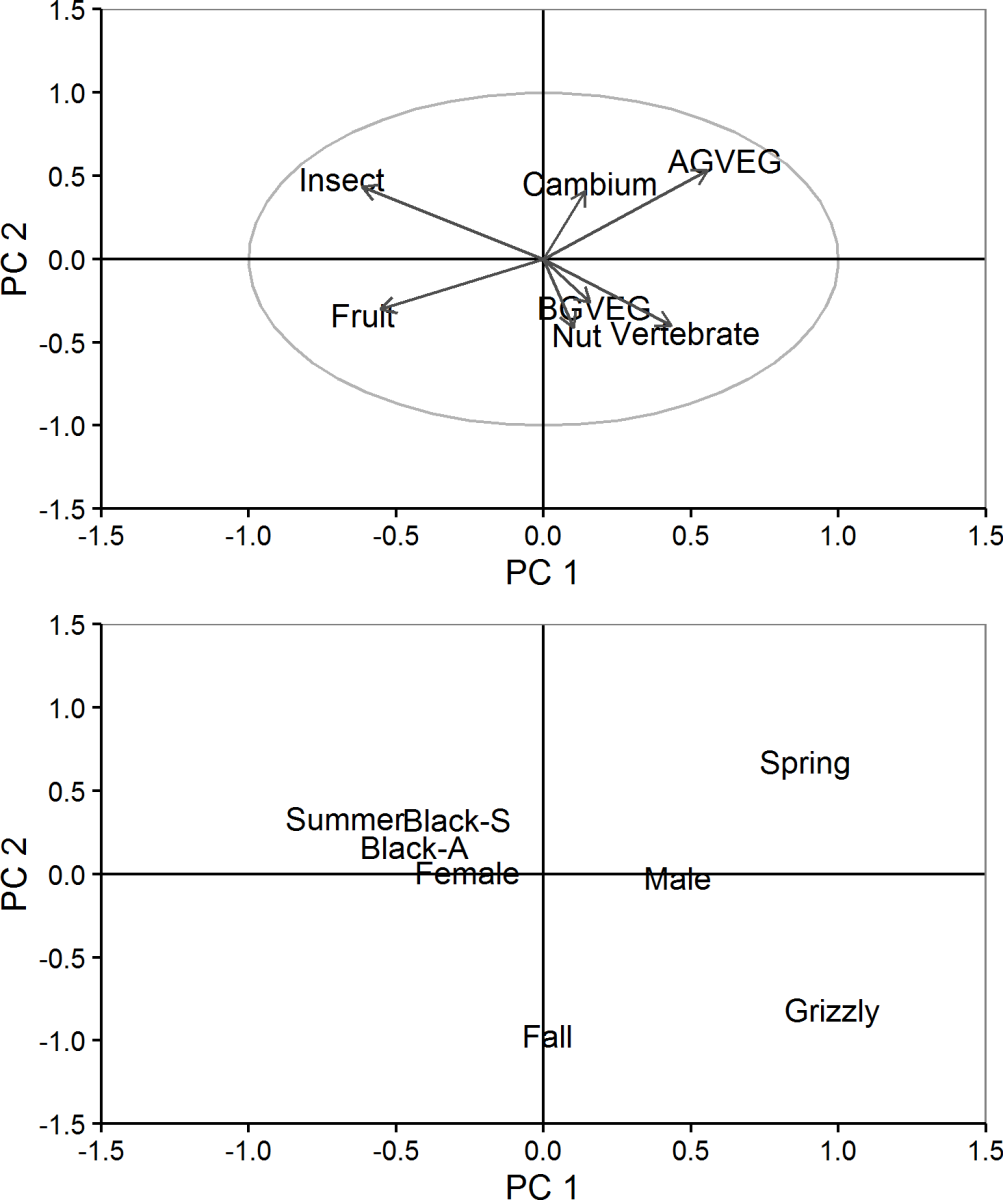
Scatter plots of the first two principal components (PC) constructed from daily diets of grizzly and black bears, Grand Teton National Park and vicinity, Wyoming, USA, 2004–2006. Top graph illustrates active variables, which were the daily percentages of total energy obtained from 7 food types (e.g., above-ground vegetation [AGVEG], below-ground vegetations [BGVEG]). Bottom graph illustrates supplementary variables of species (grizzly, sympatric black [black-S], allopatric black [black-A]), sex, and season (spring [1 May–30 Jun], summer [1 Jul–20 Aug], fall [21 Aug–31 Oct]).

Summarizing by food type, bears consumed 1–6 types/day with an average of 2.9. Bears consumed AGVEG during 86% of days and insects during 83% of days, making these two food types the most common. Fruits were consumed during 55% of days and vertebrates were consumed on 36% of days. The remaining food types were consumed on ≤11% of days. Converting daily consumption of food types into percentage of digestible energy, and using PCA on the main seven food types (excluding fungi), seven dimensions were generated. The first two and five dimensions explained 42% and 87% of the variation, respectively. The first two dimensions separated vertebrate from insect consumption, AGVEG from fruit consumption, grizzly bears from black bears, and all three seasons from one another (Fig 2). Using HCPC analyses, 14 daily diet types were identified (Table 3). We detected homogenous diets for all food types except cambium, which provided a maximum of only 15% of daily digestible energy on any bear-day. All other food types provided a maximum of 95–100% of daily energy. We detected 8 mixed diets in which digestible energy was comprised of 2 or more primary food types. Mean number of samples obtained/day was similar among diet types (Table 3) and did not differ between homogenous and mixed diet types (*t* = 0.76, *P* = 0.45). Three homogenous diet types showed no association with GPS fix interval, and the other three were split between positive and negative associations with fix interval (Table 4). These results indicate that bear-days with lower food diversity were not simply a product of lower sampling.

**Table 3:** Mean estimated percentages of daily digestible energy provided by seven primary food types within 14 diets of grizzly and black bears identified by principal components and cluster analyses of field data, Grand Teton National Park, Wyoming, USA, 2004–2006. Homogenous diets were dominated by one particular food type, whereas mixed diets were composed of 2 or more principal food types. Within these categories, diets are ordered by number of bear-days observed.

				Mean percent of daily digestible energy by food type						
Category	Daily diet type	No. bear-days	Mean no. samples/day^a^	AGVEG^b^	BGVEG^c^	Cambium	Fruit	Nut	Insect	Vertebrate
Homogeneous	Insect	45	3.9	4	0	0	13	0	82	1
	AGVEG	40	4.7	86	0	0	0	0	13	1
	Fruit	30	4.4	4	1	0	78	1	16	0
	Vertebrate	26	5.5	8	0	0	2	0	5	85
	Nut	12	6.0	2	0	0	1	91	5	1
	BGVEG	7	5.2	10	79	0	2	1	5	3
Mixed	Insect-AGVEG	35	3.6	33	0	0	5	0	60	1
	Fruit-insect	20	5.7	8	1	0	43	1	40	8
	AGVEG-vertebrate	18	5.5	53	0	0	0	1	13	32
	Insect-vertebrate	10	3.4	8	0	0	2	0	59	31
	BGVEG mixed	9	4.6	10	47	0	6	0	14	22
	AGVEG-fruit	8	3.8	41	0	0	51	0	5	3
	Cambium mixed	7	2.8	54	0	10	0	0	27	9
	Nut mixed	7	5.2	5	3	0	14	59	5	14

^a^ Sum of scat percent volumes and scat equivalent percentages assigned to foods identified by feeding sign

^b^ Above-ground vegetation

^c^ Below-ground vegetation

**Table 4:** Positive (+) and negative (-) associations of the 14 identified daily diet types with supplementary categorical variables for season, sex, and species (χ^2^ tests) and supplementary continuous variables for body mass and GPS fix interval (*t* tests), Grand Teton National Park, Wyoming, USA, 2004–2006. The number of symbols corresponds to *P*: single (0.01 ≤ *P* ≤ 0.05), double (0.001≤ *P* ≤ 0.01), triple (*P* ≤ 0.001).

			Season			Sex		Species				
Category	Daily diet type	No. bear-days	Spring	Summer	Fall	F	M	Grizzly bear	Black bear (sympatric)	Black bear (allopatric)	Body mass	Fix interval
Homogeneous	Insect	45	-	+++	-	+	-	---	++		---	
	AGVEG^a^	40	+++	--	---	---	+++					
	Fruit	30	---		+++			--		++	--	++
	Vertebrate	26		---	++	--	++	+++	---		+++	-
	Nut	12	-	--	+++	-	+					
	BGVEG^b^	7			++	+	-	+++	--		++	-
Mixed	Insect-AGVEG	35		+	--			---	+		---	++
	Fruit-insect	20	---	+++								
	AGVEG-vertebrate	18	+++		---							
	Insect-vertebrate	10			-							
	BGVEG mixed	9				+	-	+++	--		+++	--
	Nut mixed	7			++			+				
	AGVEG-fruit	8			++							
	Cambium mixed	7										

^a^ Above-ground vegetation

^b^ Below-ground vegetation

Foods and food types varied in their nutritional content (S1 Table). Based on published data, animal foods were highly digestible with high mean protein and lipid content, and were highest in energy content per unit on a dry-matter basis (Table 1). On a fresh matter basis, insects provided more energy per unit than did vertebrates, due to their lower moisture content. Although non-digestible content was highest in nuts, this deficiency was offset by the highest mean lipid content, ranking them among the highest energy foods on a dry-matter and fresh-matter basis. Energy values of all other foods were much lower than those of animal foods or nuts, due to their substantial proportions of non-digestible matter. Similar in their proportions of protein and lipid, total energy of these remaining foods was primarily determined by their carbohydrate content. On a fresh matter basis, fruits, BGVEG, and AGVEG had the highest energy content. Although cambium and fungi had slightly higher dry-matter energy content compared with these three food types, their fresh-matter energy ranked lowest due to high moisture content.

**Fig 3:**
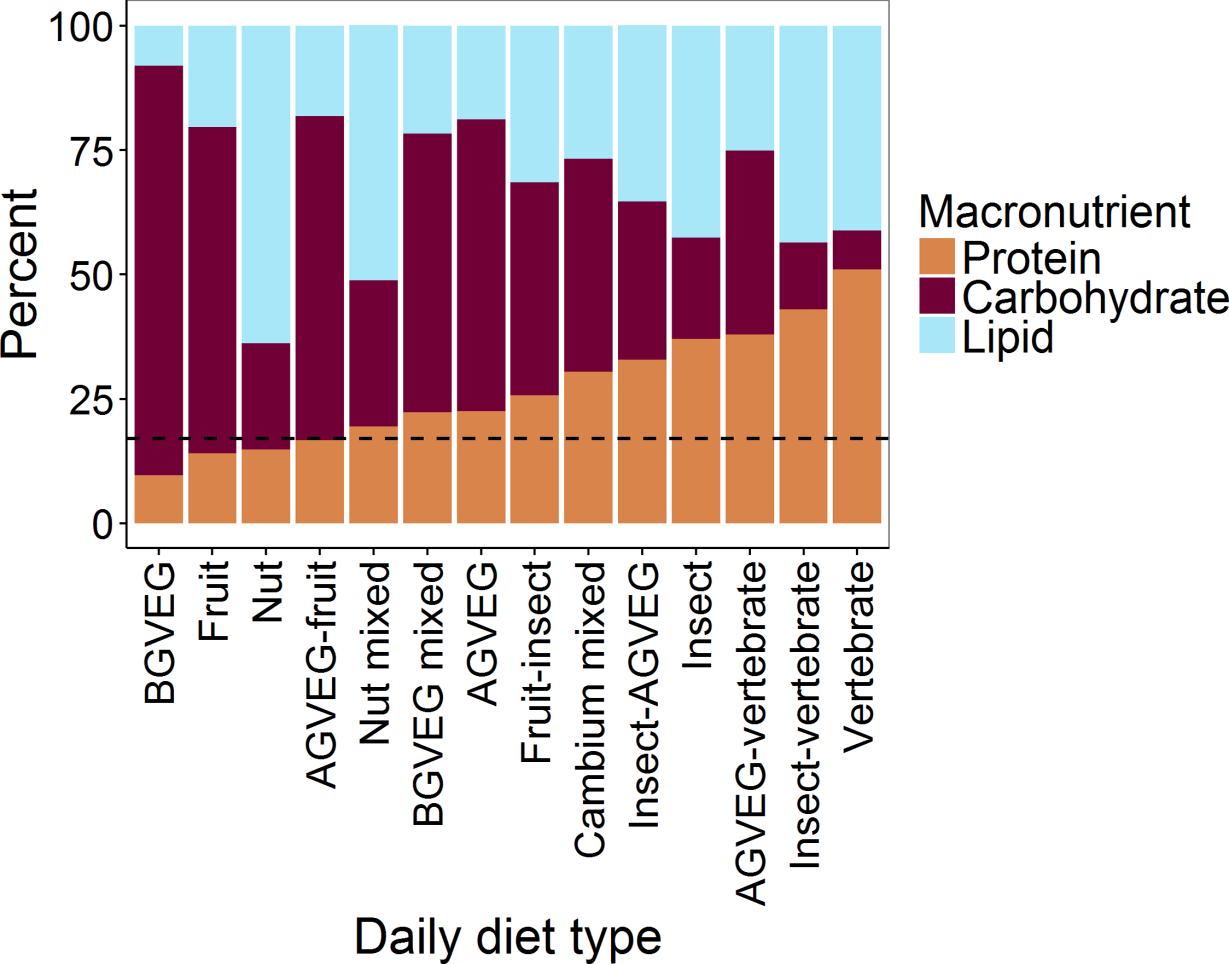
Mean estimated macronutrient content, as a percentage of daily digestible energy, for the 14 daily diet types of grizzly and black bears identified by principal component and cluster analyses of foraging data collected during site visits of bear locations during a 24-hour period, Grand Teton National Park and vicinity, Wyoming, USA, 2004–2006. Foods were categorized into 7 primary food types (e.g., above-ground vegetation [AGBVEG], below-ground-vegetation [BGVEG]). The dashed line depicts the optimal protein level (17 ± 4%) that has been shown to maximize body mass gain per unit of energy intake [8].

Mean daily percentages of macronutrients varied among observed diet types (Fig 3). One homogenous diet (BGVEG) resulted in estimated protein levels well below the optimum of 17 ± 4% based on [8]. The BGVEG diet was positively associated with fall, grizzly bears, and body mass; and was negatively associated with sympatric black bears. This homogenous diet accounted for 3% of bear-days.

Four diet types (two homogenous and two mixed) resulted in estimated mean daily protein levels within the optimal range. The fruit, nut, and nut-mixed diets were associated with fall, whereas the fruit-insect diet was associated with summer. The nut diet was positively associated with males. The fruit diet was positively associated with allopatric black bears, and was negatively associated with grizzly bears and body mass. The nut mixed diet was positively associated with grizzly bears. Together, these 4 diets accounted for 21% of days.

One homogenous and two mixed diets resulted in estimated mean daily protein levels above, but relatively close to the optimal. The BGVEG mixed diet showed a strong positive relationship with grizzly bears and body mass, and was positively associated with females. The AGVEG diet was positively associated with spring and males. The fruit-insect diet was positively associated with summer. Together, these 3 diets accounted for 25% of days.

The remaining 6 diet types (2 homogenous and 4 mixed) resulted in estimated mean daily protein levels well above the optimum, and all but one included substantial amounts of animal foods. These diets were associated with several seasons. The cambium mixed diet showed no association with sex, species, or body mass, perhaps owing to its relative rarity. The insect diet was positively associated with sympatric black bears and females, and was negatively associated with grizzly bears and body mass. The vertebrate diet showed a strong positive relationship with grizzly bears and body mass, and a negative relationship with sympatric black bears. It was also positively associated with males. Neither the AGVEG-vertebrate nor the insect-vertebrate diets were associated with either sex or species, but the AGVEG-vertebrate diet was strongly associated with spring. Together, these 6 diets types accounted for 51% of days.

**Fig 4:**
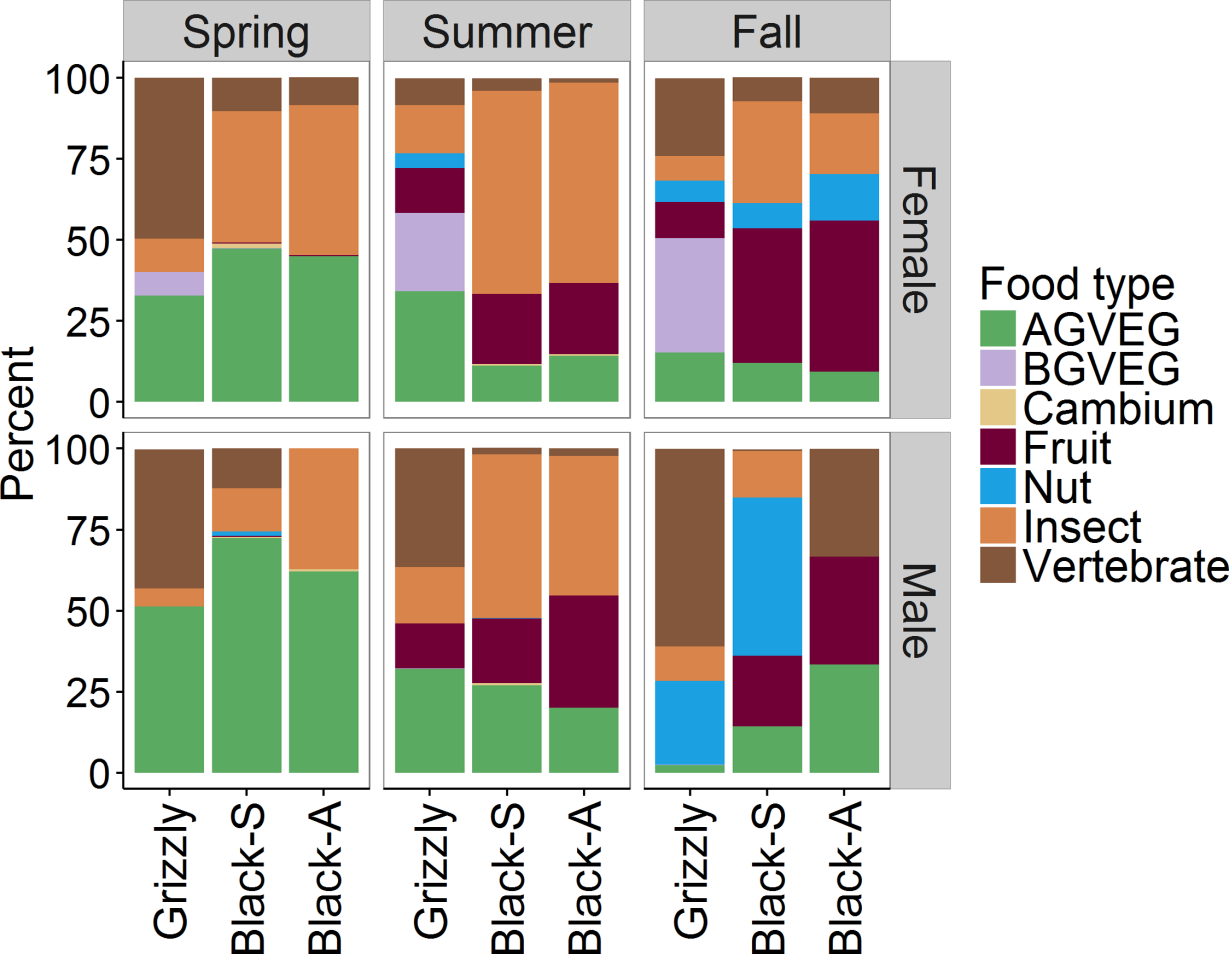
Mean estimated percentage of daily digestible energy provided by seven primary food types (e.g., above-ground vegetation [AGBVEG], below-ground-vegetation [BGVEG]) among grizzly, sympatric black (black-S), and allopatric black (black-A) bears, as documented from site visits to bear locations during a 24-hour period, Grand Teton National Park and vicinity, Wyoming, USA, 2004–2006.

Averaged by season, daily consumption of food types differed by bear species (Fig 4). Animal matter provided an average of 46–47% of the daily digestible energy for both grizzly and black bears, but vertebrates dominated the grizzly diet and insects dominated in the black bear diet. Fruits were equally important to grizzly and black bears during summer, but were less important in fall diets of grizzly bears. BGVEG provided considerable energy to female grizzly bears, but provided little energy to any other class of bear.

**Fig 5:**
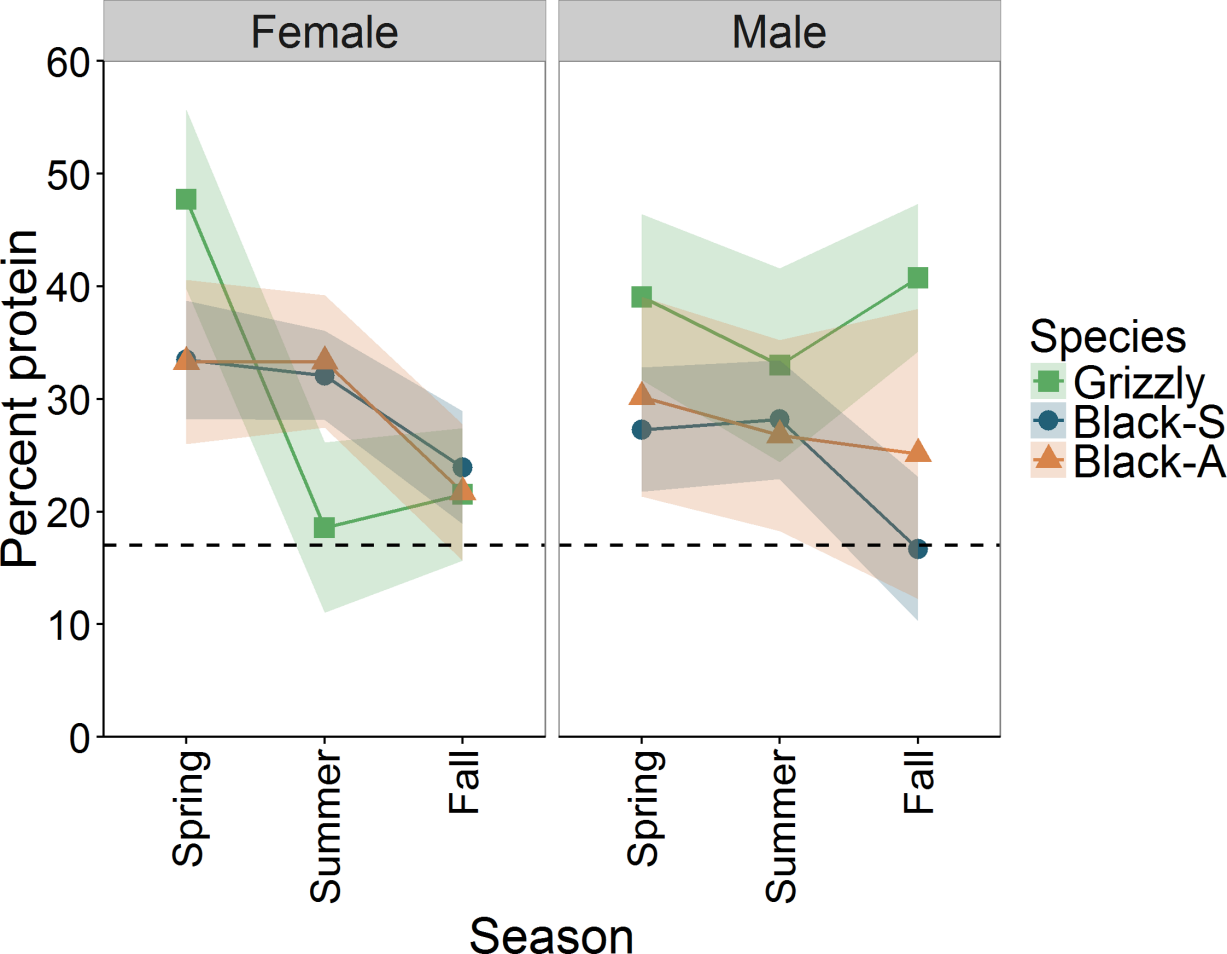
Model-predicted mean percentage of protein (±95% confidence interval) within the daily digestible energy consumed by grizzly, sympatric black (Black-S), and allopatric black (Black-A) bears, by season and sex, Grand Teton National Park and vicinity, Wyoming, USA, 2004–2006. The dashed line depicts the optimal protein level (17 ± 4%) that has been shown to maximize body mass gain per unit of energy intake [8].

Based on a mixed-effects model, mean daily percent protein varied by season (F_2,225_ = 16.75, P < 0.001), species-sex category (F_5,31_ = 4.75, P = 0.003), and season × species-sex category (F_10,225_ = 4.23, *P* < 0.001). Averaging across species-sex categories, mean daily protein intake was 7–10% greater during spring compared with summer and fall (*P* < 0.001), but we detected no difference between summer and fall (*P* = 0.06). Averaging across seasons, mean daily protein was 8–13% greater for male grizzly bears compared with all other species-sex categories (*P* ≤ 0.02). On a daily basis, all classes exceeded the optimal protein intake of 17 ± 4% during spring (Fig 5). Male grizzly bears far exceeded optimal protein during all seasons. Black bears came closest to achieving the optimal daily protein balance during fall, and grizzly bear females came closest during summer.

**Fig 6:**
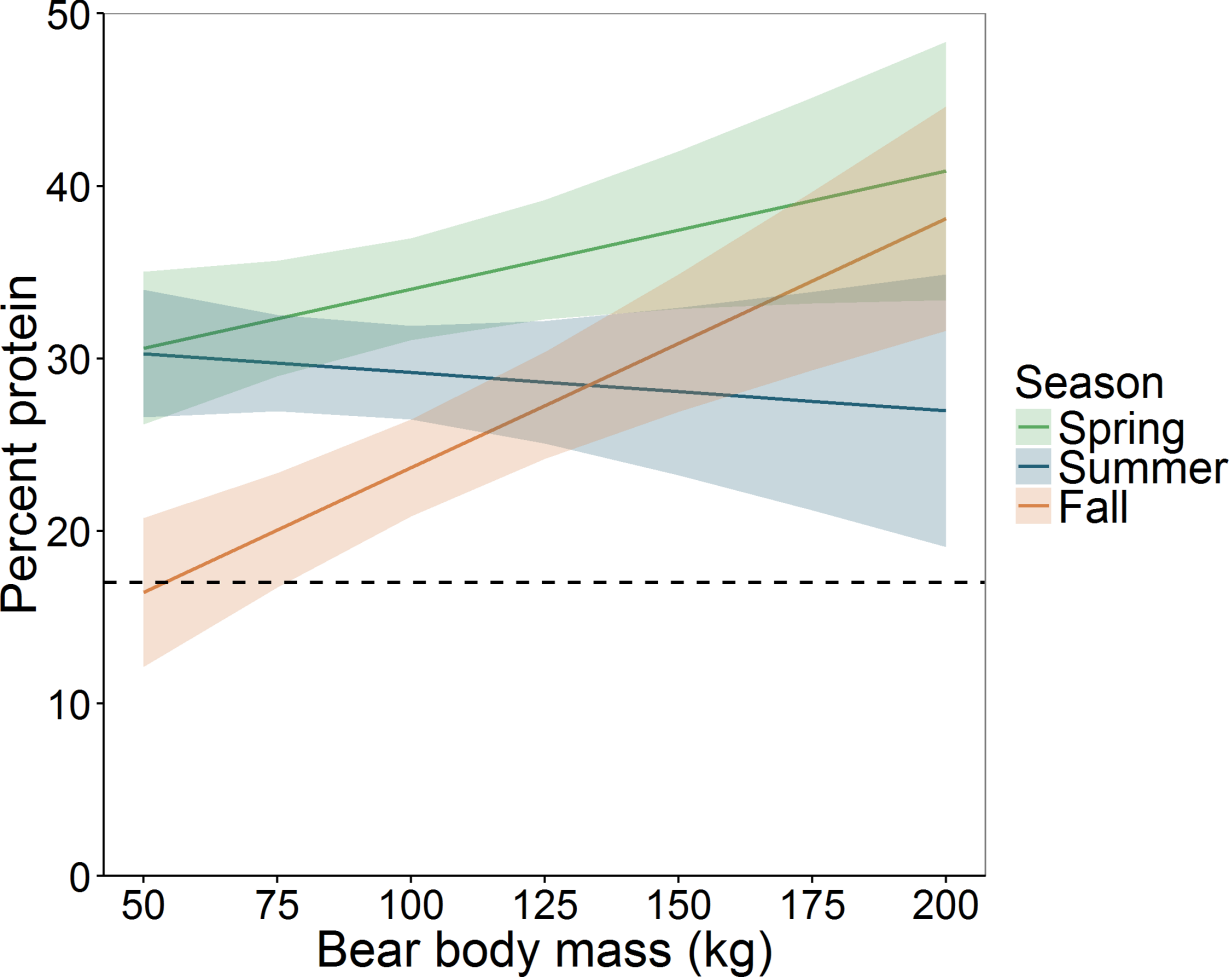
Model-predicted relationship between body mass and mean percentage of protein (± 95% CI) within the daily digestible energy consumed by bears (species combined), by season, Grand Teton National Park and vicinity, Wyoming, USA, 2004–2006. The dashed line depicts the optimal protein level (17 ± 4%) that has been shown to maximize body mass gain per unit of energy intake [8].

Pooling all bears, mixed-effects modeling also indicated that mean daily percent protein varied by season (F_2,233_ = 15.97, *P* < 0.001), body mass (F_1,35_ = 9.26, *P* < 0.004), and season × body mass (F_2,233_ = 8.64, *P* < 0.001). During fall, mean daily protein intake increased substantially with body mass, from approximately 16% for a 50-kg bear to 38% for a 200-kg bear (Fig 6). We also observed an increase over this range of body mass during spring, however 95% CIs overlapped. During summer, mean daily protein intake was similar to that of fall, but no effect of body mass was evident.

## Discussion

Under the constraints of the natural environment in our study area, grizzly and black bears were rarely able to achieve the optimal balance of protein, relative to carbohydrates and lipids, in their daily diets as observed among captive bears fed ad libitum [8]. Only 4 of the 14 daily diet types provided protein within the optimal range, and these diets accounted for only 21% of observed bear-days. As predicted, bears exceeded optimal protein consumption during spring; however, counter to our prediction, protein levels were higher than optimal for most bears during other seasons as well.

Bears were able to optimally balance macronutrients by feeding on diets dominated by whitebark pine nuts (a near-optimal food on its own) and fruit, and by mixing these energy-rich foods with protein-rich insects and AGVEG. Similar mixing of vegetation and fruit has been observed in wild black howler monkeys (*Alouatta pigra*) that were observed to maintain a relatively consistent level of daily protein intake even when non-protein energy intake varied in response to the amount of fruits consumed [34]. As predicted, the homogenous fruit diet was negatively associated with body mass, consistent with previous findings that large-bodied bears are incapable of gaining weight on fruit alone. But, the other optimal diets showed no association with body mass, indicating that all bears took advantage of optimal food combinations when seasonally available. Food mixing was also evident in various diets that resulted in slightly elevated daily protein levels.

Despite the observation that larger bears can be constrained from achieving mass gain when feeding exclusively on poorly digestible vegetation [12], the AGVEG diet type showed no association with species or body mass, thus even large-bodied bears spent at least some days feeding nearly exclusively on grasses and forbs. This diet type was strongly associated with spring, prior to the time when most energy-rich plant foods became available, providing some explanation for its consumption. Given that AGVEG-vertebrate was the other daily diet strongly associated with larger-bodied grizzly bears during spring, we propose that they might minimize loss of body mass on days when energy-rich vertebrate foods are unavailable by consuming a near macronutrient-balanced diet, even if its energy content is insufficient to meet basal metabolic needs. Similarly, the near optimal balance of the AGVEG diet supports the observation of Noyce and Garshelis [11] that black bears are capable of gaining weight while feeding primarily on vegetation.

Although ursids have evolved various traits associated with increased herbivory, they are equally efficient at digesting protein as obligate carnivores, such as felids and canids [35]. With adequate intake, bears are capable of gaining mass across a wide range of dietary protein intake [7, 8]. However, efficiency and rate of mass gain fall sharply as percent protein decreases below the optimal, but decreases more gradually as percent protein increases above the optimal. For example, bears experienced a 25% decline in the rate of mass gain when dry-matter protein content exceeded the optimal by 28%, but experienced the same decline in mass gain when dry-matter protein content fell below the optimum by only 12% [8]. Thus, when unable to optimize protein intake, consuming protein in excess of the optimum is an energetically superior trade-off compared with consuming little to no protein. In addition, even while gaining fat, bears can lose lean body mass feeding on low-protein, high-energy diets, such as those dominated by fruits [14, 36]. Our results were consistent with these findings as 9 of 14 daily diet types provided protein in excess of the optimal level, and these diets accounted for 76% of observed bear-days. In contrast, only 1 of 14 diets provided protein below the optimum, accounting for only 3% of bear-days. Indeed, bears consumed AGVEG and/or insects during nearly all days, even when feeding heavily on carbohydrate-rich fruits and BGVEG, perhaps as a strategy to boost protein levels.

Protein intake far exceeding the optimal was most notably associated with consumption of vertebrates, predominantly ungulates. As predicted, these diets were strongly associated with grizzly bears and greater body mass. Highly digestible vertebrate foods, such as ungulates and fish, are among the most energy- and nutrient-dense foods available to bears [20] and it is their consumption that creates the nutritional opportunity for achievement of large body size [12]. Thus, given the nutritional benefits of a large ungulate, coupled with only a moderate decline in the efficiency of converting the food to body mass, it seems logical that bears would concentrate their foraging on vertebrates whenever possible. We further suggest that feeding on ungulates, even exclusively, is more energetically rewarding than abandoning possession of a carcass to seek additional foods for a mixed, optimally-balanced diet. In the Greater Yellowstone Ecosystem (GYE), bears obtain ungulate meat through predation and scavenging, including usurping carcasses from other bears, wolves (*Canis lupus* [37]), and cougars (*Puma concolor* [38]), and competition at carcasses can be intense. It is unlikely that the increased metabolic efficiency achieved through diet mixing could compensate for probable loss of access to a highly valuable carcass once left behind. These circumstances are in marked contrast to those observed by Robbins et al. [7] in Alaska. They found that brown bears suspended foraging on vertebrates to feed on seasonally abundant berries, presumably to optimize protein intake. However, unlike ungulates in the GYE, the vertebrates in that case were abundant, predictable, and replenishing runs of salmon (*Oncorhynchus* spp.). Similar to our results, wild mountain gorillas (*Gorilla beringei*), have been observed to prioritize energy over protein balance when leaves are the major dietary item [39].

Insect-dominated diets also resulted in higher-than-optimal protein intake. These diets were more common among black bears than grizzly bears and were negatively associated with body mass. Several studies have reported the importance of insects, particularly ants, to black bears [40, 41]. In addition, Swenson et al. [42] reported that ants accounted for approximately 20% of annual digestible energy of brown bears in Scandinavia. Averaged across the year, our results indicate insects (predominantly ants) accounted for 12% of the daily digestible energy of grizzly bears in our study area, and 41% of the daily digestible energy of black bears. Despite their frequent consumption by bear species throughout the world, ants and other insects are still generally regarded as alternative foods; opportunistically found and consumed in large quantities only when other, presumably superior foods, such as vegetation or mast, are scarce. Noyce et al. [40] offered an alternative view. Reporting distinct seasonality, large volumes ingested, and preference for a few species, they concluded that ant foraging within some black bear populations was both directed and highly selective, similar to that observed among obligate myrmecophages. In our study, insects were consumed on the vast majority of bear-days by both grizzly and black bears. Insect and mixed insect diets were particularly important among the smaller-bodied black bears and female grizzly bears. Our literature review indicated that, gram for gram, fresh-matter energy of ants was substantially greater than that of vertebrates. The natural state of colony-nesting insects is difficult to replicate and it is not surprising that insect foods have not been a focus in studies of captive bear foraging strategies. However, direct observations of wild bears indicate that they feed only briefly at each ant colony, often leaving many ants uneaten; this strategy likely maximizes energy intake per unit handling time as ants quickly disperse upon disturbance [40, 42]. Thus, a relatively small energetic reward obtained from each excavation is likely counterbalanced by the ubiquity of the resource [41] and the nominal exertion needed to obtain the food. Anecdotal evidence suggests that black bears are capable of deriving sufficient energy to meet their metabolic needs from ants. Based on analysis of stomach contents, assumed to represent only a few hours of foraging time, Noyce et al. [40] and Auger et al. [41] estimated that ants provided 575 and 695 kcal, respectively, to two black bears. This intake is roughly half of the daily requirement for a 50-kg bear. Auger et al. [41] suggested that black bears might rival ant specialist as ant predators after documenting ant numbers in single scats similar to the entire daily intake of anteaters (suborder Vermilingua). But, larger bears may be constrained from gaining weight on insects due to the limitation of intake rate, similar to vegetation and fruit. This is supported by our findings that insects were often consumed nearly exclusively by black bears but usually were part of mixed diets in grizzly bears.

Contrary to our prediction, diets of allopatric black bears were far more similar to those of sympatric black bears than those of grizzly bears. Free from direct competition with grizzly bears, neither male nor female allopatric black bears consumed vertebrates or other foods in proportions similar to grizzly bears. This suggests that, even in the ungulate-rich environment of the GYE, black bears largely adhere to the typical plant- and insect-dominated foraging strategy documented throughout their range, with a spike in vertebrate foraging associated with availability of neonate ungulates during spring [9]. The similarity in sympatric and allopatric black bear diets strengthens the notion that insects are a staple food of black bears.

Whether derived from vertebrates or insects, we estimated that animal foods accounted for nearly half of daily digestible energy of both grizzly bears and black bears in our study area. For grizzly bears, our estimates of animal food consumption generally agreed with previous studies in the GYE based on stable isotope analyses [43, 44, 45]. For black bears, our results were consistent with Jacoby et al. [43], who found that assimilation of animal matter was similar for sympatric black bears and all grizzly bear sex-age classes (~45%), except adult males (~79%). However, our estimates for animal consumption by black bears were generally higher than those obtained by Fortin et al. [44] and Schwartz et al. [45], who found that black bears assimilated approximately half the amount of animal protein in their diet compared with grizzly bears. The inconsistencies among these studies may be explained by regional differences in diets as observed by Mealy [26], or the relatively small sample sizes for black bears in the studies of Fortin et al. [44] and Schwartz et al. [45]. Nonetheless, our observed seasonal trends in daily protein consumption matched monthly isotope-derived estimates of animal consumption by Schwartz et al. [45], which showed peaks in May and Oct among grizzly bears and in August among black bears.

Besides vertebrates, whitebark pine nuts were the other high-energy bear food available in our study area. Because nuts provide near-optimal levels of protein, and are high in lipids, it is not surprising that bears of both species and all body masses consumed nut-dominated diets. However, we did observe a sex difference in consumption rates, perhaps signaling effects of intraspecific competition. Overall, nuts accounted for approximately 14% and 18% of the daily digestible energy of grizzly bears and black bears during fall, respectively. Among grizzly bears, nuts provided 25% of the daily digestible energy to males, but only 7% to female. Similarly, they provided 39% to male black bears, but only 10% to female black bears. Blanchard and Knight [46] postulated that adult males might displace other cohorts from productive stands. Our results support the idea that competition may influence consumption of this high-energy food, but because grizzly bears obtain most nuts by excavating red squirrel (*Tamiasciurus hudsonicus*) caches [47], we suspect that male dominance is exhibited at individual caches, rather than at the stand level, similar to what is observed around ungulate carcasses. Our data also suggest that direct competition between grizzly bears and black bears might be curtailed by their differential methods of foraging. Based on feeding sign, we found that grizzly bears obtained 97% of their nut energy from digging in caches, whereas sympatric black bears obtained 51% of their nut energy from caches and 49% by harvesting cones from trees. Even more extreme, we observed that allopatric black bears obtained 100% of their nut energy by harvesting cones from trees, however this was based on a smaller sample than that of sympatric black bears.

The value of whitebark pine nuts to bears, particularly grizzly bears, is a topic of substantial interest because infestations of mountain pine beetle (*Dendroctonus ponderosae*) have caused considerable mortality of cone-producing trees within the GYE since the early 2000s [48]. Our data on the relative value of nuts may be germane to our understanding of the role of this species for several reasons. First, whitebark pine habitat encompassed about 10% of our study area, similar to its proportion within the entire GYE (14% [49]). Second, adjusting for our sample size of bear-days/year, mean cone production for our study period was 7.9 cones/tree and median count was 8 cones/tree, comparable to the long-term median estimated during 1980–2014 (8.2 [IGBST, unpublished data]). Third, although significant beetle-caused tree mortality had occurred in some sections of the GYE by the end of our study period in 2006, relatively little mortality had occurred within our GTNP study area by that time [48]. Thus, our study provides a valuable benchmark regarding the energetic value of whitebark pine nuts prior to the full onset of its decline. Whereas whitebark pine nuts were a major food during fall for both species, averaging across years, they provided less than half of the daily digestible energy among male bears of both species, and less than one quarter of the daily digestible energy among females during the fall season in GTNP. Among all classes of bears except male sympatric black bears, vertebrates and insects, taken together or even separately, ranked higher in contribution to daily digestible energy during fall than did whitebark pine nuts. Nonetheless, although sample sizes were inadequate for annual estimates, the contribution of whitebark pine nuts was much greater during 2006, a year of high cone production, similar to previous observations [47].

Our study establishes a new method for quantifying foraging habits and, to our knowledge, presents the first estimates of relative macronutrient consumption by bears in the wild. The demographic specificity of the information we obtained surpasses that of typical food habit studies based on analysis of scat contents. Whereas demographic differences in diet can be discerned using stable isotope analyses, our approach provides a more comprehensive assessment of total dietary intake. Nonetheless, our intensive, time-consuming field methods meant that sample sizes were limited for several species, sex, and season categories, perhaps limiting inference to the populations at large. Our methods had additional limitations. For example, protein content decreases and fiber content increases in many plant foods during the growing season [11, 26], but published data on food composition were inadequate to represent such trends in our analysis. When consuming large mammals or abundant salmon, studies (e.g., [50]) have shown that black and grizzly bears show some selectivity in their consumption of proteinaceous parts (i.e., muscle, skin, and viscera) versus fat tissue (e.g., blubber), however this type of selectivity was largely indiscernible in the field. Therefore, we acknowledge that some vertebrate-dominated diets may not have deviated from the optimal protein content as much as we estimated, if bears selectively consumed fat tissues. Nonetheless, as Coogan et al. [15] showed, the lipid content associated with ungulate carcasses is insufficient to provide an optimal balance alone. Additionally, although bears may preferentially feed on fat tissue upon the first feeding, it is unlikely they abandon energy- and protein-rich remains once fatty tissues have been depleted.

In summary, our data indicate that bears in our study were largely unable to select foods to optimize macronutrient intake on a daily basis, and were constrained by body size, season, and competition. When unable to optimize intake, bears usually opted for the energetically superior trade-off of over-consuming protein, and generally avoided protein deficits by feeding on mixed diets. Although most daily diets were not optimized for maximum mass gain, we should not assume they were inadequate for either body maintenance or growth. We were unable to quantify actual daily energy intake (i.e., total kcal consumed) on an absolute or relative basis, therefore we could not determine if bears achieved adequate intake of calories on a daily basis. But, based on bioelectrical impedance measures of captured bears, Schwartz et al. [45] estimated that both grizzly and black bears experienced monthly fat gains during most of the active season in the GYE. Among grizzly bears, percent body fat declined between May and June, but increased each month from June to October. Among black bears, percent body fat was relatively constant between May and July, and then increased monthly from July to October. Interpreting our results together with those of Schwartz et al. [45], we propose the following depiction of the seasonal energetics of grizzly bears and black bears in our study. During spring, when both species largely subsisted on low-energy plant foods, smaller-bodied black bears were probably able to maintain both fat stores and lean body mass. In contrast, due to the constraint of intake rate, larger-bodied grizzly bears likely continued to utilize stored body fat gained prior to hibernation, while maintaining lean body mass due to the high protein content of the diet. Large deficits in calories were minimized by both species with the consumption of neonate vertebrates and winter-killed ungulates. Once more energy-rich plant foods became available during summer and fall, bears were able to gain both fat and lean body mass, by mixing plant and animal foods. Even smaller-bodied black bears, energetically able to persist on fruit-dominated diets, often selected mixed diets, minimizing the need to catabolize lean body mass to fulfill amino acid requirements, as observed among bears in British Columbia [14]. Whereas male bears appeared capable of outcompeting female conspecifics for some high-energy foods, direct competition between grizzly bears and black bear was minimized due to differences in food selection and methods of accessing foods.

## Supporting Information

S1 Appendix Field methods and quantification of relative daily intake of foods by grizzly and black bears, based on site visits of GPS-monitored individuals, Grand Teton National Park, Wyoming, USA, 2004–2006. (PDF)

S1 Table Estimated percent composition of food items based on published literature sources, used to estimate diet and relative macronutrient intake of grizzly and black bears during 24-hour periods, based on site visits of GPS-monitored individuals during 2004–2006, Grand Teton National Park, Wyoming, USA. (PDF)
