# Supplementary material for: Diet and Macronutrient Optimization in Wild Ursids: A Comparison of Grizzly Bears with Sympatric and Allopatric Black Bears
Source: PLoS One. 2016 May 18;11(5):e0153702. doi: 10.1371/journal.pone.0153702 (PMC4871523; doi:10.1371/journal.pone.0153702)
Supplement: S1 Appendix — (PDF) [file pone.0153702.s001.pdf]

S1 Table. Estimated percent composition of food items based on published literature sources, used to estimate diet and relative macronutrient intake of grizzly and black bears during 24-hour periods, based on site visits of GPS-monitored individuals during 2004–2006, Grand Teton National Park, Wyoming, USA.

			Percent composition (fresh matter basis)					
Food type	Sub-type	Genus or lowest taxa identified	Moisture	Protein	Carbohydrate	Lipid	Non-digestable[a]	Sources (or substitutions[b])
AGV[c]	Fern ally	*Equisetum*	74.8	5.9	10.1	0.7	8.5	8, 11, 12, 28, 29
AGV	Fern ally	*Equisetum arvense*	74.8	5.9	10.1	0.7	8.5	8, 11, 12, 28, 29
AGV	Fern ally	*Lycopodium*	74.8	5.9	10.1	0.7	8.5	mean AGV-fern ally
AGV	Forb	*Agoseris*	76.8	5.4	9.6	1.2	7.0	4, 14, 33
AGV	Forb	*Angelica arguta*	10.2	11.3	47.2	7.1	24.2	17
AGV	Forb	*Aquilegia*	75.4	4.0	10.8	1.4	8.4	mean AGV-forb
AGV	Forb	*Aster conspicuum*	76.8	5.6	4.6	0.6	12.4	32
AGV	Forb	*Balsamorhiza saggittata*	75.4	4.0	10.8	1.4	8.4	30, 44, 45
AGV	Forb	*Campanula rotundifolia*	75.4	4.0	10.8	1.4	8.4	mean AGV-forb
AGV	Forb	*Castilleja*	75.4	4.0	10.8	1.4	8.4	mean AGV-forb
AGV	Forb	*Castilleja miniata*	75.4	4.0	10.8	1.4	8.4	mean AGV-forb
AGV	Forb	*Cerastium*	73.3	11.3	8.3	1.0	6.0	12
AGV	Forb	*Chimaphila*	75.4	4.0	10.8	1.4	8.4	mean AGV-forb
AGV	Forb	*Cirsium*	89.5	1.3	5.3	0.3	3.5	6, 8, 29, 44
AGV	Forb	*Claytonia*	76.8	6.9	10.3	1.1	5.0	8, 29
AGV	Forb	*Claytonia lanceolata*	74.5	1.9	15.5	0.2	7.9	29, 45
AGV	Forb	*Clematis*	75.4	4.0	10.8	1.4	8.4	mean AGV-forb
AGV	Forb	*Collomia*	75.4	4.0	10.8	1.4	8.4	mean AGV-forb
AGV	Forb	*Corallorhisa maculata*	75.4	4.0	10.8	1.4	8.4	mean AGV-forb
AGV	Forb	*Epilobium*	81.4	2.1	8.7	1.6	6.1	20, 42, 44
AGV	Forb	*Epilobium angustiflium*	81.4	2.1	8.7	1.6	6.1	20, 42, 44
AGV	Forb	*Eriogonum*	76.8	2.9	11.0	1.3	8.0	4, 44
AGV	Forb	*Erythronium grandiflorum*	44.5	6.2	27.7	2.8	18.9	8, 28, 30, 45
AGV	Forb	*Fritillaria pudica*	74.8	2.4	15.4	0.2	7.3	20, 45

S1 Table (continued).

Food type	Sub-type	Genus or lowest taxa identified	Percent composition (fresh matter basis) Moisture	Protein	Carbohydrate	Lipid	Non-digestable[a]	Sources (or substitutions[b])
AGV	Forb	*Galium triflorum*	76.8	1.7	2.4	2.7	16.4	25, 49
AGV	Forb	*Hedysarum occidentale*	50.3	5.9	16.4	0.8	26.6	7, 8, 28
AGV	Forb	*Helianthella uniflora*	76.8	2.4	11.3	1.4	8.2	34
AGV	Forb	*Heracleum lanatum*	95.0	0.7	2.2	0.2	1.9	8, 20, 25, 28, 29
AGV	Forb	*Hieracium*	75.4	4.0	10.8	1.4	8.4	mean AGV-forb
AGV	Forb	*Hydrophyllum*	75.4	4.0	10.8	1.4	8.4	mean AGV-forb
AGV	Forb	*Iliamna rivularis*	75.4	4.0	10.8	1.4	8.4	mean AGV-forb
AGV	Forb	*Ligusticum*	76.8	2.3	11.3	1.4	8.2	34
AGV	Forb	*Ligusticum filicinum*	76.8	2.3	11.3	1.4	8.2	34
AGV	Forb	*Lupinus*	76.8	3.8	10.5	1.3	7.6	34, 44
AGV	Forb	*Medicago sativa*	76.8	3.5	8.3	0.7	10.7	23, 39
AGV	Forb	*Osmorhiza depauperata*	76.8	3.5	11.2	0.5	8.0	8
AGV	Forb	*Pedicularis*	75.4	4.0	10.8	1.4	8.4	mean AGV-forb
AGV	Forb	*Pedicularis bracteosa*	75.4	4.0	10.8	1.4	8.4	mean AGV-forb
AGV	Forb	*Perideridia gairdneri*	75.4	4.0	10.8	1.4	8.4	mean AGV-forb
AGV	Forb	*Potentilla*	75.4	4.0	10.8	1.4	8.4	mean AGV-forb
AGV	Forb	*Potentilla gracilis*	75.4	4.0	10.8	1.4	8.4	mean AGV-forb
AGV	Forb	*Prosartes trachycarpa*	75.4	4.0	10.8	1.4	8.4	mean AGV-forb
AGV	Forb	*Pterospora*	75.4	4.0	10.8	1.4	8.4	mean AGV-forb
AGV	Forb	*Rudbeckia*	75.4	4.0	10.8	1.4	8.4	mean AGV-forb
AGV	Forb	*Rumex*	90.6	1.7	3.9	0.7	3.1	1, 20, 25, 42
AGV	Forb	*Streptopus amplexifolius*	76.8	5.1	9.8	1.2	7.1	15
AGV	Forb	*Taraxacum*	85.6	2.7	7.6	0.7	3.5	1, 25, 42
AGV	Forb	*Thalictrum*	76.8	4.0	10.9	1.5	6.8	1, 8, 34
AGV	Forb	*Tragopogon*	75.4	4.0	10.8	1.4	8.4	44

S1 Table (continued).

Food type	Sub-type	Genus or lowest taxa identified	Percent composition (fresh matter basis) Moisture	Protein	Carbohydrate	Lipid	Non-digestable[a]	Sources (or substitutions[b])
AGV	Forb	*Tragopogon dubius*	75.4	4.0	10.8	1.4	8.4	44
AGV	Forb	*Trifolium*	85.9	3.6	5.2	0.3	5.1	4, 8, 29, 32, 36
AGV	Forb	Unidentified Apiaceae	75.4	4.0	10.8	1.4	8.4	mean AGV-forb
AGV	Forb	Unidentified Asteraceae	75.4	4.0	10.8	1.4	8.4	mean AGV-forb
AGV	Forb	Unidentified forb	75.4	4.0	10.8	1.4	8.4	mean AGV-forb
AGV	Forb	*Valerian*	75.4	4.0	10.8	1.4	8.4	mean AGV-forb
AGV	Forb	*Viola*	75.4	4.0	10.8	1.4	8.4	mean AGV-forb
AGV	Forb	*Wyethia*	75.4	4.0	10.8	1.4	8.4	mean AGV-forb
AGV	Graminoid	*Agropyron*	35.1	6.9	22.6	1.9	33.5	33, 34
AGV	Graminoid	*Bromus*	35.1	6.5	22.8	1.9	33.7	33, 34, 44
AGV	Graminoid	*Calamagrostis*	35.1	9.6	24.8	2.5	28.0	8, 29, 32
AGV	Graminoid	*Calamagrostis canadensis*	35.1	9.6	24.8	2.5	28.0	8, 29, 32
AGV	Graminoid	*Calamagrostis rubescens*	35.1	9.6	24.8	2.5	28.0	8, 29, 32
AGV	Graminoid	*Carex*	74.8	4.6	8.8	0.6	11.2	8, 12, 15, 33, 34
AGV	Graminoid	*Deschampsia*	46.0	6.8	18.3	1.7	27.2	15, 26
AGV	Graminoid	*Elymus*	35.1	5.6	23.1	1.9	34.2	15, 34
AGV	Graminoid	*Festuca*	35.1	9.6	13.9	3.7	37.7	8, 28, 30, 45
AGV	Graminoid	*Melica*	35.1	7.5	22.2	1.9	33.4	mean AGV-graminoid
AGV	Graminoid	*Phleum*	10.2	6.2	47.0	2.1	34.5	38
AGV	Graminoid	*Phleum pratense*	10.2	6.2	47.0	2.1	34.5	38
AGV	Graminoid	*Poa*	64.5	9.9	7.7	1.4	16.5	12, 26, 29, 43, 44
AGV	Graminoid	*Scirpus*	5.1	0.4	13.1	0.0	81.4	45
AGV	Graminoid	*Stipa*	35.1	6.3	22.9	1.9	33.8	34
AGV	Graminoid	Unidentified graminoid	35.1	7.5	22.2	1.9	33.4	mean AGV-graminoid
AGV	Woody plant	*Geranium viscosissimum*	76.8	3.5	3.2	2.4	14.1	25

S1 Table (continued).

Food type	Sub-type	Genus or lowest taxa identified	Percent composition (fresh matter basis) Moisture	Protein	Carbohydrate	Lipid	Non-digestable[a]	Sources (or substitutions[b])
AGV	Woody plant	*Salix*	76.8	3.5	3.2	2.4	14.1	mean AGV-woody plant
AGV	Woody plant	*Spiraea*	76.8	3.5	3.2	2.4	14.1	mean AGV-woody plant
AGV	Woody plant	Unidentified shrub	76.8	3.5	3.2	2.4	14.1	mean AGV-woody plant
BGV[c]	Aquatic	*Potamogeton*	7.2	13.5	37.5	2.2	39.7	24, 31
BGV	Forb	*Claytonia*	74.5	1.9	15.5	0.2	7.9	29, 45
BGV	Forb	*Lomatium*	37.2	4.1	34.2	0.9	23.6	8, 29, 45
BGV	Forb	*Perideridia*	44.1	2.7	42.0	0.3	11.0	8, 16, 29, 45
BGV	Forb	*Polygonum*	79.2	1.9	12.4	0.2	6.3	8, 12, 29, 49
BGV	Forb	Unidentified root	53.2	3.1	25.9	0.8	17.1	mean BGV
BGV	Graminoid	*Melica*	50.3	2.2	39.9	0.4	7.2	8, 29
Cambium		*Abies lasiocarpa*	90.1	1.2	4.2	0.2	4.3	mean cambium (10, 14, 21, 38)
Cambium		*Picea engelmannii*	90.1	1.2	4.2	0.2	4.3	mean cambium (10, 14, 21, 38)
Cambium		*Pinus contorta*	87.9	3.6	6.6	0.6	1.3	10, 13
Cambium		*Populus tremuloides*	92.0	0.2	5.0	0.5	2.3	20
Cambium		Unidentified cambium	90.1	1.2	4.2	0.2	4.3	mean cambium (10, 14, 21, 38)
Fruit		*Amelanchier alnifolia*	78.4	1.0	14.4	0.9	5.3	8, 19, 46
Fruit		*Arctostaphylos uva-ursi*	75.0	0.7	10.4	1.3	12.7	8, 19
Fruit		*Crataegus douglasii*	70.1	1.3	21.0	1.2	6.5	1, 6, 8, 19, 46
Fruit		*Fragaria virginiana*	59.1	3.4	24.6	2.7	10.3	1, 8, 18, 19, 29
Fruit		*Juniperus communis*	28.5	2.7	45.9	7.5	15.5	6, 8
Fruit		*Lonicera involucrata*	8.4	7.2	66.5	4.7	13.3	8, 18, 40
Fruit		*Lonicera utahensis*	8.4	7.2	66.5	4.7	13.3	8, 18, 40
Fruit		*Mahonia repens*	12.5	10.7	52.4	6.3	18.2	8, 18
Fruit		*Prunus virginiana*	28.2	7.3	39.7	4.2	20.7	8, 18, 40, 46
Fruit		*Rhamnus alnifolia*	51.9	3.6	29.6	2.9	12.1	mean fruit

S1 Table (continued).

Food type	Sub-type	Genus or lowest taxa identified	Percent composition (fresh matter basis) Moisture	Protein	Carbohydrate	Lipid	Non-digestable[a]	Sources (or substitutions[b])
Fruit		*Ribes*	83.7	1.0	9.0	1.0	5.3	1, 8, 19, 29, 42
Fruit		*Rosa*	29.7	6.1	35.6	3.6	25.0	1, 6, 18, 19, 42
Fruit		*Rubus*	72.5	2.2	15.5	1.7	8.0	1, 6, 8, 18, 19, 21, 40, 42, 46
Fruit		*Rubus parviflora*	72.5	2.2	15.5	1.7	8.0	1, 6, 8, 18, 19, 21, 40, 42, 46
Fruit		*Shepherdia canadensis*	81.0	1.6	11.3	0.6	5.5	8, 19, 28
Fruit		*Sorbus scopulina*	73.7	1.4	19.0	1.2	4.9	8, 46
Fruit		*Symphoricarpos*	3.8	6.1	60.2	4.9	25.1	8, 18, 40
Fruit		*Vaccinium*	73.4	1.9	18.1	1.5	5.0	1, 8, 19, 21, 28, 40, 42, 46
Fruit		*Vaccinium cespitosum*	73.4	1.9	18.1	1.5	5.0	1, 8, 19, 21, 28, 40, 42, 46
Fruit		*Vaccinium membranaceum*	73.4	1.9	18.1	1.5	5.0	1, 8, 19, 21, 28, 40, 42, 46
Fruit		*Vaccinium scoparium*	73.4	1.9	18.1	1.5	5.0	1, 8, 19, 21, 28, 40, 42, 46
Fungi		Unidentified mushroom	89.5	1.9	5.4	0.5	2.7	1, 29, 43
Insect		Formicidae	61.7	17.1	5.2	9.6	6.4	2, 32, 35, 41, 47
Insect		Unidentified insect	61.7	15.0	1.5	7.3	14.5	50
Insect		Unidentified insect larva	40.3	13.3	35.8	7.7	2.9	3, 5, 48
Insect		Vespidae	61.7	18.3	1.4	8.2	10.4	50
Nut		*Pinus albicaulis*	5.9	11.7	15.8	25.1	41.5	8, 22, 29, 45
Vertebrate	Large	*Alces alces*	71.1	19.4	0.0	8.4	1.2	27
Vertebrate	Large	*Bison bison*	71.1	19.4	0.0	8.4	1.2	27
Vertebrate	Large	*Bos taurus*	70.3	16.6	0.0	9.0	4.2	9, 36
Vertebrate	Large	*Cervus elaphus*	71.1	19.9	0.0	8.2	0.8	27, 29
Vertebrate	Large	*Cervus elaphus (neonate)*	77.7	18.0	0.0	1.4	2.9	Odocoileus (neonate)
Vertebrate	Large	*Odocoileus*	65.3	16.6	0.0	13.9	4.2	9, 28, 36
Vertebrate	Large	*Odocoileus (neonate)*	77.7	18.0	0.0	1.4	2.9	9
Vertebrate	Large	*Odocoileus hemionus*	65.3	16.6	0.0	13.9	4.2	9, 28, 36

S1 Table (continued).

Food type	Sub-type	Genus or lowest taxa identified	Percent composition (fresh matter basis) Moisture	Protein	Carbohydrate	Lipid	Non-digestable[a]	Sources (or substitutions[b])
Vertebrate	Large	Unidentified Cervidae	71.3	18.5	0.0	8.0	2.3	mean Cervidae
Vertebrate	Large	Unidentified large mammal	71.1	18.3	0.0	8.2	2.4	mean large mammal
Vertebrate	Large	*Ursus americanus*	71.1	18.3	0.0	8.2	2.4	mean large mammal
Vertebrate	Large	*Ursus arctos*	71.1	18.3	0.0	8.2	2.4	mean large mammal
Vertebrate	Small	*Ondatra zibethicus*	82.0	13.1	0.0	2.1	2.8	9
Vertebrate	Small	*Tamiasciuris*	75.5	16.2	0.2	3.6	4.4	mean small mammal (9, 36)
Vertebrate	Small	*Thomomys talpoides*	75.5	16.2	0.2	3.6	4.4	mean small mammal (9, 36)
Vertebrate	Small	Unidentified Aves	65.4	24.7	0.0	6.4	3.4	mean Aves
Vertebrate	Small	Unidentified Phasianidae	65.4	24.7	0.0	6.4	3.4	9
Vertebrate	Small	Unidentified small mammal	75.5	16.2	0.2	3.6	4.4	mean small mammal (9, 36)
Vertebrate	Small	Unidentified Aves (egg)	72.0	13.4	2.1	12.5	0.0	42
Vertebrate	Small	Unidentified vertebrate	72.5	18.0	0.1	6.1	3.3	mean small mammal (9, 36)

[a] Non-digestible matter included fiber, ash, and minerals.

[b] When values for some or all constituents were unavailable, mean values for similar food types were substituted as shown.

[c] AGV (above-ground vegetation)

[d] BGV (below-ground vegetation)

S1 Table (continued).

Sources

1. Arnason T, Hebda RJ, Johns T. 1981. Use of plants for food and medicine by native peoples of eastern Canada. Can J Botany 59: 2189–2325.
2. Auger J, Ogborn GL, Pritchett CL, Black HL. 2004. Selection of ants by the American black bear (*Ursus americanus*). Western North Amer Nat 64: 166–174.
3. Banjo AD, Lawal OA, Songonuga EA. 2006. The nutritional value of fourteen species of edible insects in southwestern Nigeria. Afr J Biotech 5: 298–301.
4. Barnett JK, Crawford JA. 1994. Pre-laying nutrition of sage grouse hens in Oregon. J Range Manage 47: 114–118.
5. Bukkens SD. 1997. The nutritional value of edible insects. Ecol Food Nutr 36: 287–319.
6. Cicnjak LD, Huber D, Roth HU, Ruff RL, Vinovrski Z. 1987. Food habits of brown bears in Plitvice Lakes National Park, Yugoslavia. Int Conf Bear Res Manage 7: 221–226.
7. Coogan SC, Nielson SE, Stenhouse GB. 2012. Spatial and temporal heterogeneity creates a “brown tide” in root phenology and nutrition. ISRN Ecol: 618257.
8. Craighead JJ, Sumner J, Mitchell JA. 1995. The grizzly bears of Yellowstone; their ecology in the Yellowstone ecosystem, 1959–1992. Island Press.
9. Dierenfeld ES, Alcorn HL, Jacobsen KL. 2002. Nutrient composition of whole vertebrate prey (excluding fish) fed in zoos. USDA Agri Res Serv.
10. Dilbone M, Turner NJ, von Aderkos P. 2013. Lodgepole pine cambium (*Pinus contorta* Dougl. ex Loud. var. latifolia Engelm. ex S. Wats.): a springtime first peoples’ food in British Columbia. Ecol Food Nutr 52: 130-147.
11. Garmo TH. 1986. Chemical composition and *in vitro* digestibility of indigenous mountain pasture plants in different plant groups (preliminary report). Rangifer 6: 14–22.
12. Gathier G. 1993. Feeding ecology of nesting greater snow geese. J Wildl Manage 57: 216–223.
13. Gottsfeld LMJ. 1995. The role of plant foods in traditional Wet’suwet’en nutrition. Ecol Food Nutr 34: 149–169.
14. Gregg MA, Barnett JK, Crawford JA. 2008. Temporal variation in diet and nutrition of preincubating greater sage-grouse. Rangeland Ecol Manage 61: 535–542.
15. Hanley TA, McKendrick JD. 1983. Seasonal changes in chemical composition and nutritive value of native forages in a spruce-hemlock forest in southeastern Alaska. UDSA For Serv Res Pap PNW-312.
16. Kaldy MS, Johnston A, Wilson DB. 1980. Nutritive value of Indian bread-root, squaw-root, and Jerusalem artichoke. Econ Bot 34: 352–357.

S1 Table (continued).

17. Kim E, Choi J, Yeo I. 2012. The effects of *Angelica keiskei Koidz* on the expression of antioxidant enzymes related to lipid profiles in rats fed a high fat diet. Nutr Res Pract 6: 9–15.
18. King TR, McClure HE. 1944. Chemical composition of some American wild feedstuffs. J Agr Res 69: 33–46.
19. Kuhnlein HV. 1989. Nutrient values in indigenous wild berries used by the Nuxalk people of Bella Coola, British Columbia. J Food Compos Anal 2: 28–36.
20. Kuhnlein HV. 1990. Nutrient values in indigenous wild plant greens and roots used by the Nuxalk people of Bella Coola, British Columbia. J Food Compos Anal 3: 38–46.
21. Landers JL, Hamilton RJ, Johnson AS, Marchinton RL. 1979. Food and habitat of black bears in southeastern North Carolina. J Wildl Manage 43: 143–153.
22. Lanner RM, Gilbert BK. 1994. Nutritive value of whitebark pine seeds, and the question of their variable dormancy. In: USDA Gen Tech Rep INT-GTR-309, pp. 206–211.
23. Lawrence LM, Coleman RJ. 2000. Choosing hay for horses. Univ Ky Coop Ext Serv Rep ID-146.
24. Linn JG, Goodrich RD, Otterby DE, Meiske JC, Staba EJ. 1975. Nutritive value of dried or ensiled aquatic plants. II. Digestibility by sheep. J Anim Sci 41: 610–615.
25. Lukač B, Kramberger B, Meglič V, Verbič J. 2012. Importance of non-leguminous forbs in animal nutrition and their ensiling properties: a review. Žemdirbystė (Agriculture) 99: 3–8.
26. Mathiesen SD, Utsi THA. 2000. The quality of the forage eaten by Norwegian reindeer on South Georgia in summer. Rangifer 20: 17–24.
27. Mattson DJ, Barber K, Maw R, Renkin R. 2004. Coefficients of productivity for Yellowstone's grizzly bear habitat. USFWS Biol Sci Rep 2002–0007.
28. McLellon BN, Hovey FW. 1995. The diet of grizzly bears in the Flathead River drainage of southeastern British Columbia. Can J Zool 73: 704–712.
29. Mealey SP. 1975. The natural food habits of free ranging grizzly bears in Yellowstone National Park, 1973–1974. Thesis, Mont St Univ.
30. Mullin WJ, Peacock S, Loewen DC, Turner NJ. 1998. Macronutrient content of yellow glacier lily and balsamroot; root vegetables used by indigenous peoples of northwestern North America. Food Res Int 30: 769–775.
31. Muztar AJ, Slinger SJ, Burton JH. 1978. Chemical composition of aquartic macrophytes I. Investigation of organic constituents and nutritional potential. Can J Plant Sci 58: 829–841.

S1 Table (continued).

32. Noyce KV, Kannowski PB, Riggs MR. 1997. Black bears as ant-eaters: seasonal associations between bear myrmecophagy and ant ecology in north-central Minnesota. Can J Zool 75: 1671–1686.
33. Paulsen HA. 1969. Forage values on a mountain grassland-aspen range in western Colorado. J Range Manage 22: 102–107.
34. Pederson JC, Harper KT. 1979. Chemical composition of some important plants of southeastern Utah summer ranges related to mule deer reproduction. Great Basin Nat 39: 122–128.
35. Pekar S, Mayntz D. 2014. Comparative analysis of the macronutrient content of central European ants (Formicidae): implications for ant-eating predators. J Insect Physiol 62: 32–38.
36. Pritchard GT, Robbins CT. 1990. Digestive and metabolic efficiencies of grizzly and black bears. Can J Zool 68: 1645–1651.
37. Radwan MA. 1969. Chemical composition of the sapwood of four tree species in relation to feeding by the black bear. For Sci 15: 11–16.
38. Scribner FL. 1889. Grasses of mountain meadows and deer parks. Bull Agr Exp Station Univ Tenn 4: 59–68.
39. Sniffen CJ, O'Connor JD, Van Soest PJ, Fox DG, Russell JB. 1992. A net carbohydrate and protein system for evaluating cattle diets: II. carbohydrate and protein availability. J Anim Sci 70: 3562-3577.
40. Spinner GP, Bishop JS. 1950. Chemical analysis of some wildlife foods in Connecticut. J Wildl Manage 14: 175–180.
41. Swenson JE, Jansson A, Riig R, Sandegren F. 1999. Bears and ants: mymecophagy by brown bears in central Scandinavia. Can J Zool 77: 551–561.
42. U.S. Department of Agriculture. 2014. USDA national nutrient database for standard reference, release 27, http://www.ars.usda.gov/ba/bhnrc/ndl.
43. van Arendonk JJCM, Poorter H. 1994. The chemical composition and anatomical structure of leaves of grass species differing in relative growth rate. Plant Cell Environ 17: 963–970.
44. Wagoner SJ. 2011. The effects of spring cattle grazing on the nutritional ecology of mule deer (*Odocoileus hemionus*) in eastern Washington. Thesis, Wash St Univ.
45. Wandsnider L. 1997. The roasted and boiled: food composition and heat treatment with special emphasis on pit-hearth cooking. Univ Neb Anthrop Faculty Pub 28.
46. Wanio WW, Forbes EB. 1941. The chemical composition of forest fruits and nuts from Pennsylvania. J Agr Res 62: 627–635.

S1 Table (continued).

47. Yamazaki K, Kozakai C, Kioke S, Morimoto H, Goto Y, Furubayashi K. 2012. Myrmecophagy of Japanese black bears in the grasslands of the Ashio area, Nikko National Park, Japan. Ursus 23: 52–64.
48. Yhoung-Aree J, Puwastien P, Attig GA. 1997. Edible insects in Thailand: an unconventional protein source? Ecol Food Nutr 36: 133–149.
49. Yildirim E, Dursun A, Turan M. 2001. Determination of nutrition contents of the wild plants used as vegetables in upper Coruh Valley. Turk J Bot 25: 367–371.
50. Xiaoming C, Ying F, Hing A, Zhiyong C. 2010. Review of nutritive value of edible insects. In Durst, PB, Johnson DV, Leslie RN, Shono K (eds), Forest insects as food: humans bite back, UN Food Agri Org, pp. 85–92.
