# Supplementary material for: Diet and Macronutrient Optimization in Wild Ursids: A Comparison of Grizzly Bears with Sympatric and Allopatric Black Bears
Source: PLoS One. 2016 May 18;11(5):e0153702. doi: 10.1371/journal.pone.0153702 (PMC4871523; doi:10.1371/journal.pone.0153702)
Supplement: S1 Table — (PDF) [file pone.0153702.s002.pdf]

S1 Appendix. Field methods and quantification of relative daily intake of foods by grizzly and black bears, based on site visits of GPS-monitored individuals, Grand Teton National Park, Wyoming, USA, 2004–2006

For each bear-day, we visited all successful GPS locations collected for a 24-hour period, except for those bears with fix intervals of <1 hour, for which we visited at least one GPS fix each hour. Site visits typically occurred within a few days to 2 weeks after the GPS bear locations, with an average of 7 days. Within 20 m of each location, we searched for scats and evidence of feeding activity (e.g., grazing, excavations, carcass remains).

Approximate age of scats was evaluated by condition and moisture content. Scats that appeared too recent or too old, relative to the GPS date, were not included in analyses. Within our raw sample, number of scats collected per bear-day ranged from 0 to 24, but the count of 24 was an outlier. This bear-day was associated with a female with yearling offspring. Whereas cub scats were usually distinguishable from their mother's and were not included in analyses, scats of yearling offspring were not always distinguishable from their mother's. Therefore, we reviewed the data and found that only one grizzly bear female in our sample was accompanied by yearling offspring. This family group accounted for 4 bear-days: 2 days with lower counts of scats (n = 6), 1 day with a high count (n = 12) and the other day with the extreme high count (n = 24). For the two days with higher counts, we sub-sampled the scats (selecting 33% of scats for each trial), evaluated the resulting difference in estimated scat contents, and found little difference. This suggested that, if some portion of these scats were left by yearling offspring, they were consuming the same foods as their mother. However, we did not wish to overestimate the contribution of the foods in scats, due to an excessive number of collected scats. Therefore, for these two days with high scat counts that were associated with yearling family groups, we combined contents of all scats collected at each site and treated them as a single scat. This reduced the scat counts from 12 and 24 to 5 and 9, respectively. Our final scat sample ranged from 0 to 13 scat/day, with a mean of 2.6 (Fig. a). These numbers are consistent with Roth (1976), who documented defecation rates of brown bears in captivity and reported a range of 0 to11 scats/day, with seasonal means ranging from 2 to 7.

Our methods required that we combine observations of feeding activity with scats to estimate relative consumption of different foods and food types within a bear-day. All

observations of feeding sign at GPS bear locations were subjectively classified according to three levels of intensity:

1) Light consumption - when only a few bites of a food were likely consumed, exemplified by grazing on a few individuals plants; fruit gleaned from a few branches; an isolated excavation for insects; and feeding on an old previously-consumed carcass.

2) Moderate consumption - when many bites were likely consumed, exemplified by observation of a measurable area of grazing; disturbance of several shrubs, trees, or logs for feeding; and feeding on a small vertebrate.

3) Heavy consumption - when a large volume of a food was likely consumed, exemplified by disturbance of a large proportion of the plot area for grazing, excavation, or other feeding; and feeding on a large, fresh vertebrate.

Sometimes multiple, usually consecutive fixes were obtained at the same site. Observations of feeding sign were assigned to the first fix only, unless bears were observed to feed on a large, fresh ungulate carcass or were observed to feed at a large whitebark pine cache (with intensity values of 3). In this case, additional scat equivalents for this feeding sign were assigned to later fixes, but only if the time between locations (i.e., the time the bear stayed at the site) exceeded 3 hours.

We used intensity scores to assign a "scat equivalent" percentage to each observed food. For each food type, we calculated the proportions of 1, 2, and 3 intensity scores recorded from feeding sign. To estimate the appropriate scat equivalent percentage values, we utilized the distribution of percent dietary (i.e., corrected) volumes of foods observed, by food type, within our entire scat sample (Table a). For example, 69% of the 899 observations of above-ground feeding sign were assigned an intensity score of 1. Correspondingly, the lowest 69% of scat volumes (1492 of 2162) for above-ground vegetation ranged from <1% to 29%, with a median of 9%. Using this process, we estimated the median observed percent volumes corresponding to the three intensity values for each food type. Summarizing across food types, the mean, median and mode values were all equal, therefore we used these values for our assignment of scat equivalent percentages (i.e., %%, 30%, and 75%).

To evaluate the potential influence of these assigned values on our results, we substituted alternative values and summarized food consumption for each bear-day. First, we substituted the minimum values among the food types (i.e., 2%, 13%, and 75%). Secondly, we substituted the maximum values among the food types (i.e., 9%, 51%, and 100%).

Percentages of daily digestible energy provided by each of the 8 food types observed on a given bear-day differed by -18% to 18% comparing assigned values to alternative minimum values, and differed by -17% to 20% comparing assigned values to alternative maximum values. The median difference ranged from -1% to 2%, with most equal to 0. All distributions were centered near 0, indicating that estimates were unbiased relative to the assigned scat equivalent values (Fig. b, for example). Similarly, estimates of the percentages of daily digestible energy provided by each of the 3 macronutrients differed by -11% to 12% comparing assigned values to alternative minimum and maximum values. Median differences were all equal to 0, thus all distributions were centered at 0, indicating that estimates were unbiased relative to the assigned scat equivalent values (Fig. c).

Finally, results of mixed-effects linear regression models testing for differences in percentages of protein in daily digestible energy as a function of season and species-sex categories, and as function of season and bear body mass were virtually indistinguishable comparing analyses using our assigned scat equivalent values and analyses using the alternative minimum and maximum scat equivalent values (Tables b and c, Figs. d and e).

Literature cited

Roth, HU. Defecation rates of captive brown bears. Int. Conf. Bear Res. Manage.1976; 4: 249–253.

Table a. Frequency of intensity scores among food types observed during site visits to GPS locations of grizzly bears, and corresponding percent volumes of food types used to assign scat equivalent percentages to intensity scores.

	Frequency of observed feeding sign				Corresponding percent volumes observed in scats used to assign "scat-equivalent" percentages to observations of feeding sign						
		Proportion by intensity score				Lowest proportion (intensity 1)		Middle proportion (intensity 2)		Highest proportion (intensity 3)	
Food type	*n*	1	2	3	*n*	Range	Median	Range	Median	Range	Median
Above-ground vegetation	899	0.69	0.27	0.04	2162	<1 - 29	9	30 - 89	37	90 - 100	100
Below-ground vegetation	75	0.33	0.48	0.19	133	<1 - 17	5	18 - 65	30	66 - 97	75
Cambium	35	0.71	0.20	0.09	37	<1 - 11	5	12 - 56	30	57 - 75	75
Fruit	587	0.52	0.40	0.08	1168	<1 - 21	5	22 - 75	30	76 - 100	95
Insect	764	0.50	0.38	0.12	1133	<1 - 19	5	20 - 75	30	76 - 100	75
Nut	70	0.36	0.27	0.37	137	<1 - 36	5	37 - 75	51	76 - 100	93
Vertebrate	245	0.03	0.13	0.84	417	<1 - 3	2	4 - 25	13	26 - 100	75
					Mean		5		30		75
					Median		5		30		75
					Mode		5		30		75

Table b. Results of mixed-effects linear regression models testing for differences in percentages of protein in daily digestible energy as a function of season and species-sex categories, using assigned scat equivalent values and alternative minimum and maximum scat equivalent values.

			Assigned		Minimum		Maximum	
Parameter	Numerator df	Demonimator df	F	*P*	F	*P*	F	*P*
Intercept	1	225	1087.22	<0.001	1017.53	<0.001	1113.49	<0.001
Season	2	225	16.75	<0.001	16.08	<0.001	16.80	<0.001
Species-sex category	5	31	4.75	0.004	4.79	0.002	4.36	0.004
Season: species-sex category	10	225	4.23	<0.001	3.63	<0.001	4.72	<0.001

Table c. Results of mixed-effects linear regression models testing for differences in percentages of protein in daily digestible energy as a function of season and bear body mass, using assigned scat equivalent values and alternative minimum and maximum scat equivalent values.

			Assigned		Minimum		Maximum	
Parameter	Numerator df	Demonimator df	F	*P*	F	*P*	F	*P*
Intercept	1	233	833.83	<0.001	790.17	<0.001	878.30	<0.001
Season	2	233	15.97	<0.001	15.62	<0.001	15.62	<0.001
Body mass	5	35	9.26	0.004	10.10	0.003	8.11	0.007
Season: body mass	10	233	8.64	<0.001	8.21	<0.001	8.49	<0.001

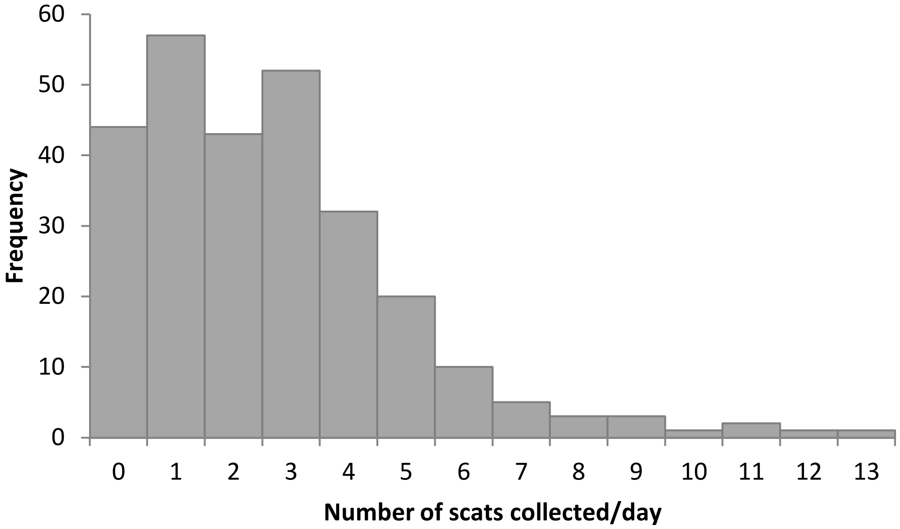

Fig. a. Frequencies of the number of scats collected/day among the 274 bear-days of documented food consumption.

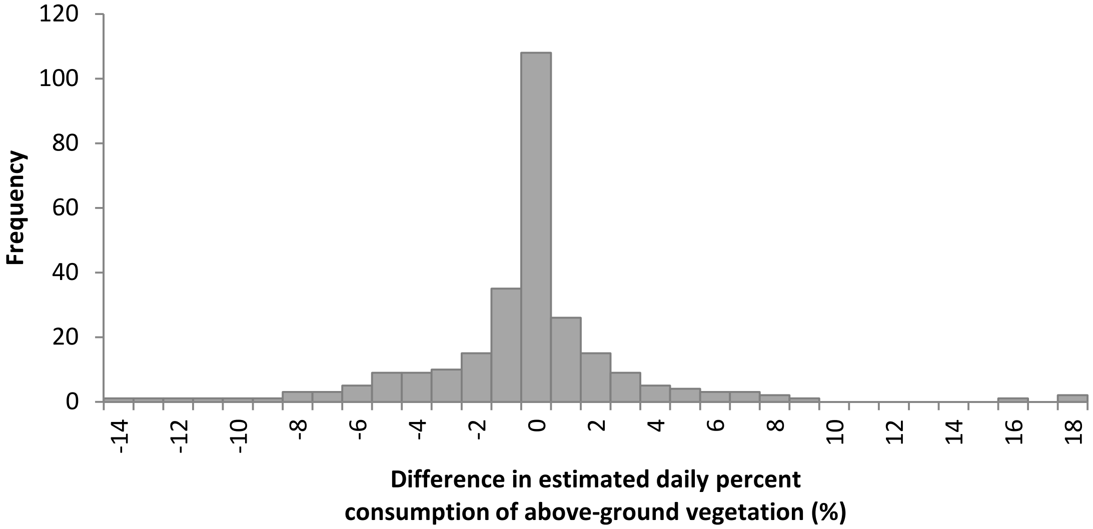

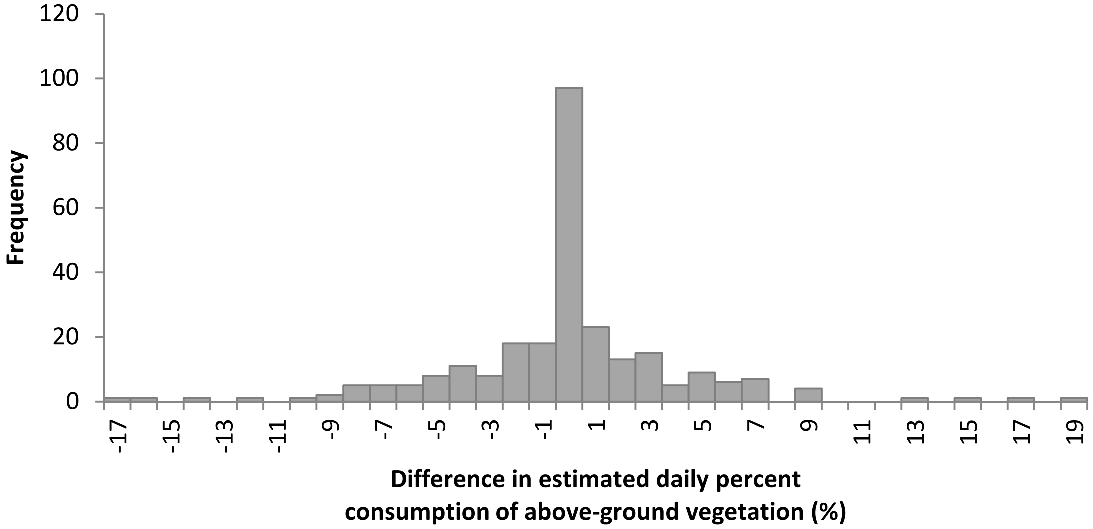

Figure b. Differences in estimates of daily consumption of above-ground vegetation during 230 bear-days when this food was consumed, comparing estimates utilizing assigned scat equivalent values for signs of feeding activity to alternative minimum scat equivalent values (top) and maximum scat equivalent values (bottom). Among food types, above-ground vegetation was consumed on the highest proportion of days (86%), but histograms for other food types were similarly unbiased.

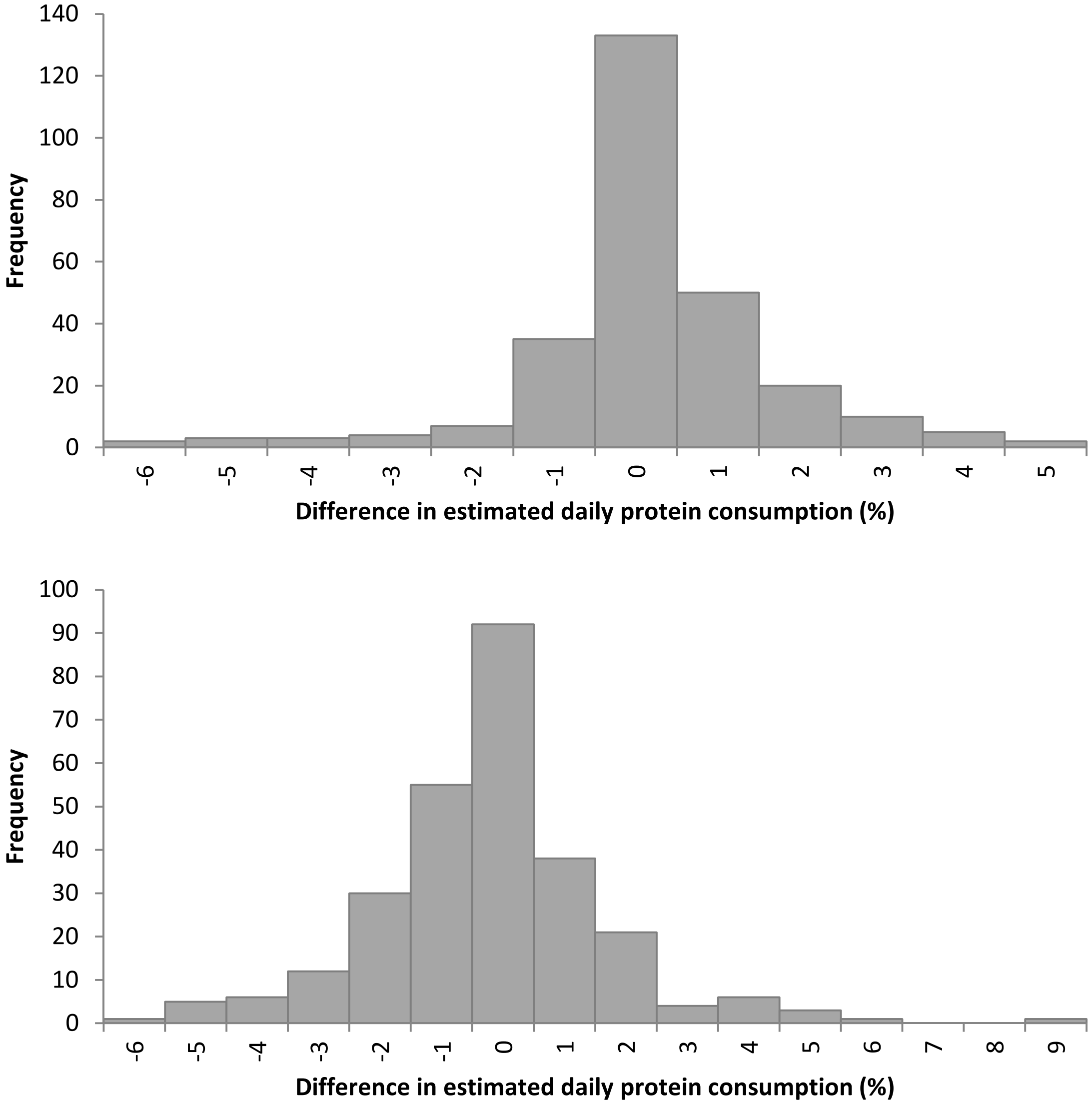

Figure c. Differences in estimates of daily consumption of protein during 274 bear-days, comparing assigned scat equivalent values to alternative minimum values (top) and maximum values (bottom). Protein consumption was a focus of our analyses, but histograms for other macronutrients were similarly unbiased.

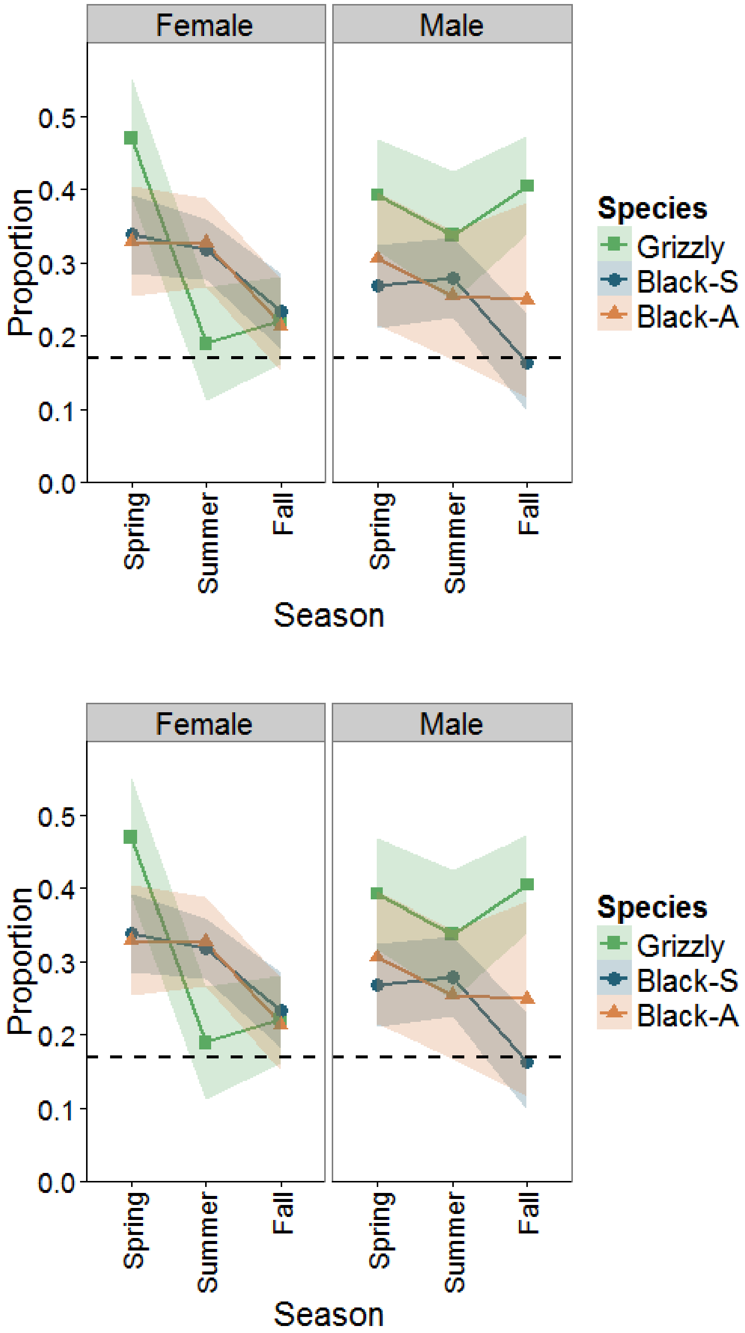

Figure d. Model-predicted mean percentage of protein in daily digestible energy (±95% confidence interval) using the alternative minimum scat equivalent values (top) and maximum scat equivalent values (bottom). These results were virtually indistinguishable from results using the assigned scat equivalent values (see results and Fig. 5 in paper).

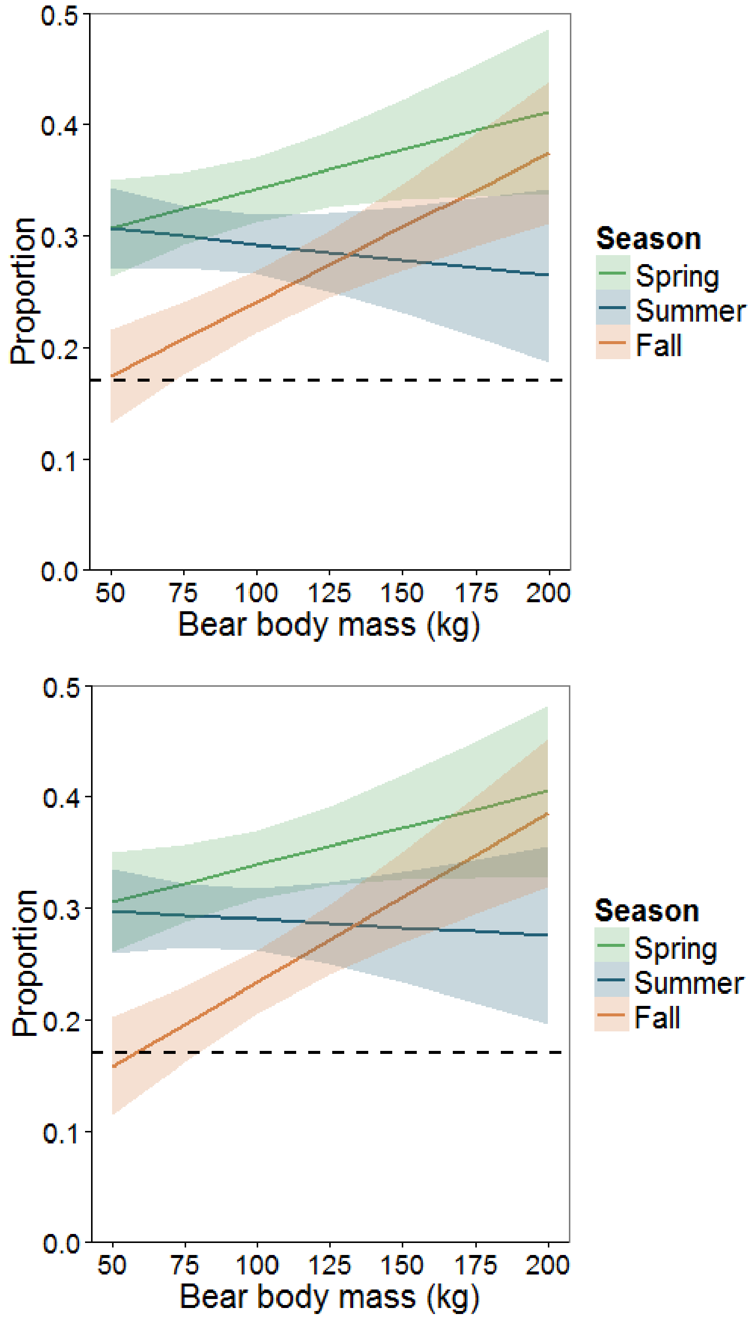

Fig. e. Model-predicted relationship between body mass and mean percent protein in daily diets of grizzly and black bears combined using the alternative minimum scat equivalent values (top) and maximum scat equivalent values (bottom). These results were virtually indistinguishable from results using the assigned scat equivalent values (see results and Fig. 6 in paper).
